# A DFT Study of Alkaline Earth Metal-Doped FAPbI_3_ (111) and (100) Surfaces

**DOI:** 10.3390/molecules28010372

**Published:** 2023-01-02

**Authors:** Maryam RaeisianAsl, Saeedeh Sarabadani Tafreshi, Nora H. de Leeuw

**Affiliations:** 1 Department of Chemistry, Amirkabir University of Technology, No. 350, Hafez Avenue, Valiasr Square, Tehran 1591634311, Iran; 2 School of Chemistry, University of Leeds, Leeds LT2 9JT, UK; 3 Department of Earth Sciences, Utrecht University, 3584 CB Utrecht, The Netherlands

**Keywords:** FAPbI_3_, perovskite solar cells, density functional theory, alkaline earth metals, doping

## Abstract

Density functional theory calculations have been performed to study the effect of replacing lead by alkaline earth metals on the stability, electronic and optical properties of the formamidinium lead triiodide (FAPbI_3_) (111) and (100) surfaces with different terminations in the form of FAPb_1-x_AE_x_I_3_ structures, where AE is Be, Mg or Ca. It is revealed that the (111) surface is more stable, indicating metallic characteristics. The (100) surfaces exhibit a suitable bandgap of around 1.309 and 1.623 eV for PbI_5_ and PbI_6_ terminations, respectively. Increases in the bandgaps as a result of Mg- and Ca-doping of the (100) surface were particularly noted in FAPb_0.96_Ca_0.04_I_3_ and FAPb_0.8_Ca_0.2_I_3_ with bandgaps of 1.459 and 1.468 eV, respectively. In the presence of Be, the band gap reduces critically by about 0.315 eV in the FAPb_0.95_Be_0.05_I_3_ structure, while increasing by 0.096 eV in FAPb_0.96_Be_0.04_I_3_. Optimal absorption, high extinction coefficient and light harvesting efficiency were achieved for plain and doped (100) surfaces in the visible and near UV regions. In order to improve the optical properties of the (111)-PbI_3_ surface in initial visible areas, we suggest calcium-doping in this surface to produce FAPb_0.96_Ca_0.04_I_3_, FAPb_0.92_Ca_0.08_I_3_, and FAPb_0.88_Ca_0.12_I_3_ structures.

## 1. Introduction

Within about ten years of first being reported, halide perovskites that can be manufactured at low temperatures with low cost [1,2,3] have emerged in optoelectronic applications with rising efficiencies from 3.8% to more than 25.7% [4]. Halide perovskites generally have the ABX_3_ formula, where A is an organic or inorganic cation such as methylammonium (MA), formamidinium (FA), or cesium (Cs), whereas B is a metal ion such as lead (Pb) or tin (Sn), and X is a halide anion (e.g., Cl, Br, I). Perovskite sensitizers have absorption coefficients of ~10^5^ cm^−1^ to enable significant light absorption, tunable bandgaps from 1.2 to over 3 eV, small exciton binding energies lower than 100 meV, and long diffusion lengths exceeding 1 μm for both holes and electrons [5,6,7,8,9,10,11,12,13]. Among the perovskite family, methylammonium lead triiodide (MAPbI_3_=MAPI) is the most widely studied material for solar cell applications [14,15], owing to its substantial absorption coefficient in the visible regime [6,16], high carrier mobility [17], and a tunable band gap energy (E_g_) [7,11]. However, although considerable research has focused on the MAPI perovskite [18,19], it has become clear that MAPI suffers from poor stability at high temperatures [3,20], under continuous light illumination [21,22] and humidity [23,24], due to the volatile nature of the MA cation [20,25,26,27]. In particular, MAPI decomposes into PbI_2_ at temperatures higher than 85 °C [18,20], and the practical application of this material is therefore limited.

**Figure 1:**
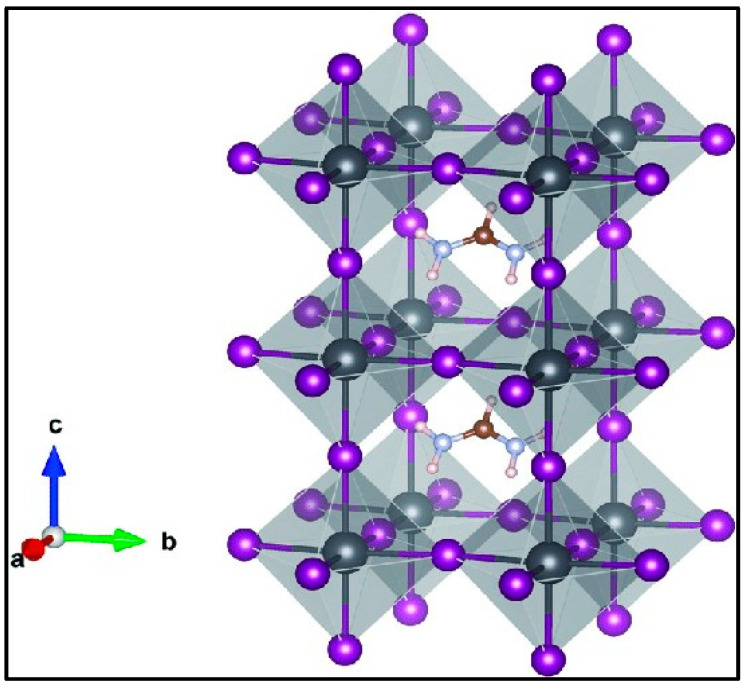
Crystal structure of cubic FAPI bulk material. Pb = grey, I = purple, C = brown, N = light blue, H = pink.

To address these issues, the formamidinium lead iodide (FAPbI_3_=FAPI) perovskite has been suggested as an alternative to MAPI, owing to its broader light absorption range toward the near-infrared region, reduced tendency to form/release volatile species, longer charge diffusion length, much improved thermal stability and potential high-efficiency in solar cells (a maximum theoretical PCE of 32.3% [28,29]) [3,30,31,32,33]. According to Goldschmidt’s tolerance factor calculations [34] and experimental results [35], at 298K [36] there are two major phases for this perovskite, namely a photo-active α-phase (black phase) and a photo-inactive hexagonal δ-phase (yellow phase) [37]. The α-phase is categorized into three classifications, i.e., a cubic (α) phase and tetragonal (β and γ) phases [35,38,39,40,41]. The crystal structure of the cubic phase of FAPI is portrayed in Figure 1, where each Pb atom is surrounded by six I atoms, four I atoms share the corner positions of the octahedron, and the organic FA cation is located in a cage surrounded by [PbX_6_] octahedra [42,43]. Despite the good performance of this material, at low temperatures (below 150 °C) [13], the desired cubic FAPI crystal (α-FAPI) with optimal band gap shows a gradual phase transformation [30] into a transparent δ-FAPI phase with an inappropriate bandgap of ~2.48 eV [44] and a one-dimensional non-perovskite crystal structure [45,46]. Such instability is caused by the larger size of FA^+^ cation and poses a challenge for practical applications of FA-based perovskites. In various studies it has been confirmed that α-FAPI perovskites can be stabilized by mixing or substituting methods: for instance, including a small number of cations with smaller ionic radii such as MA^+^, Cs^+^ and Rb^+^ at the A-site [40,46,47,48,49,50], the addition of MACl [51], introduction of silica/perovskite interfaces [52], or low concentrations of OH^−^ (strong alkaline additives [NaOH or KOH]) [53] could decrease the phase instability issue of FAPI. Additionally, it has been revealed that Br^−^ mixing is the most effective method of stabilizing the α-phase of FAPI_,_ but as Br^−^ opens the band gap, MA co-mixing is required, whereas Cs^+^ mixing contributes to prevent the decomposition of MHPs into precursors [54]. Although the Pb-based materials have unique properties, they suffer from lead toxicity. As such, many studies have focused on mixing the Pb content with appropriate elements to reduce the toxicity, whilst also providing other beneficial effects [55], e.g., remarkable optoelectronic properties or efficiency [56] of this dopant atom in the FAPI perovskite material. For example, the long-term stability is improved and decomposition prevented by partial replacement of Eu at the Pb site [57], the stability of the cubic phase is enhanced by replacing the Pb atoms in FAPI with transition metals TM [58], the toxicity of the organic-inorganic metal-halide perovskite is reduced whilst retaining the unique contribution of Pb by partial replacement of Pb by Mg [56], where stabilization of the FAPI without altering its cubic symmetry has been confirmed when small fractions up to 7% of Pb are replaced [59]. Moreover, partial replacement of Pb^2+^ with Cd^2+^ ions produces a stable Cd−I bond, which leads to an enhancement of the perovskite stability. It is worth noting that extra PbI_2_ forming from the combination of the released Pb^2+^ ions with I^−^ ions improves the stability and the PCE [60].

In different studies, it has been found that the absorption coefficient around the solar spectrum and electronic structure of germanium halide perovskites bear a close similarity to that of lead perovskites, with a small energy difference between the non-bonding orbital and antibonding orbital, but with a large energy difference compared to that of tin, showing that only tin perovskites have a low absorption coefficient [61]. The replacement of Pb by Sn has no significant effect on the effective masses, whereas mass is increased in the Ge-containing perovskites [62,63]. The Ge-containing compounds have a higher dielectric constant compared to their Pb- and Sn-containing counterparts [62]. FA_0.75_Cs_0.25_Sn_0.25_Ge_0.75_I_3_ has photovoltaic properties which are close to those of FA_0.75_Cs_0.25_Sn_0.5_Pb_0.5_I_3_ [64] with a very high photoelectric conversion efficiency, but the former does not contain toxic atoms [65]. Incorporating the highly stable Ca^2+^ and Sr^2+^ rather than the less stable Ge^2+^ and Sn^2+^ at the B-site reduces pollution and this incorporation in addition to A- and X-site incorporation is responsible for increased stability [66].

First-principles calculations are a powerful tool to investigate the optical and electronic properties of perovskite solar cell materials. In this study, we have first focused on the surface stability of four surfaces of the FAPI perovskite, i.e., the (100) surface with terminations of PbI_5_, PbI_6,_ and the (111) surface with PbI_3_ and PbI_6_ terminations. Next, following the classical notion of Goldschmidt’s rules, various amounts of alkaline earth metals Be, Mg, and Ca have been substituted into the lead sites of these surfaces, where their stabilities have been examined. In the final part of our study, the electronic properties and optical parameters are calculated for all the plain and doped surfaces.

## 2. Results and Discussion

### 2.1. Surface Structures

**Figure 2:**
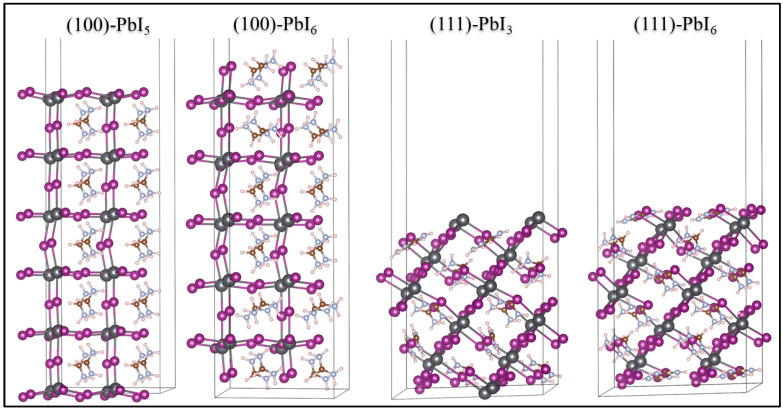
Structural geometries of the plain FAPI (100) and (111) surfaces with different terminations. The FA units are shown in a combination of H (white), N (blue), and C (brown) atoms, while I and Pb atoms are shown in purple and grey balls.

In order to gain insight into the intrinsic characteristics of FAPI-based perovskite materials, we first considered the total energy minimization of the FAPI bulk. Next, we created the surfaces from the optimized structure of the bulk to form stable (100) and (111) directions [67] with PbI_3_, PbI_5_, and PbI_6_ terminations, creating four surfaces: (100)-PbI_5_, (100)-PbI_6_, (111)-PbI_3_ and (111)-PbI_6_, all shown in Figure 2. For the surface optimizations, we kept fixed the middle bulk layers and relaxed the outer layers to provide symmetric structures.

#### 2.1.1. Stability of the Plain Surfaces

Following optimization, we calculated the surface stabilities. For this purpose, the cleavage energies, $E_{Cl}$ for each surface were calculated using the two different terminations *T*_1_ and *T*_2_ of each surface, corresponding to the PbI_3_ and PbI_6_ terminations of the (111) surface and the PbI_5_ and PbI_6_ terminations of the (100) surface, according to the equation:

(1) $$E_{Cl}^{\left(111\right),\;\left(100\right)}=\frac{E_{slab}^{unrel}\left(T_{1}\right)+E_{slab}^{unrel}\left(T_{2}\right)-NE_{Bulk}}{4A}$$

where $E_{Slab}^{unrel}\left(T_{1}\right)$ and $E_{slab}^{unrel}\left(T_{2}\right)$ are the single point energies of the unrelaxed slabs of the two terminations of each surface, *E_bulk_* is the energy of the bulk of FAPI, N is the total number of FAPI units in the slabs of both terminations together (44 in our case), and A is the surface area of the slabs. The relaxation energy $\Delta E_{Surf}^{rel}$ is then calculated for each termination of *T*_1_ and *T*_2_ separately, using Equation (2) as:

(2) $$\Delta E_{surf}^{rel\;\left(T_{1},\;T_{2}\right)}=\frac{E_{slab}^{rel}\left(T_{1},\;T_{2}\right)-E_{slab}^{unrel}\left(T_{1},\;T_{2}\right)}{2A}$$

where $E_{slab}^{rel\;\left(T_{1},T_{2}\right)}$ and $E_{slab}^{unrel\;\left(T_{1},T_{2}\right)}$ are the relaxed and unrelaxed energies of each termination *T*_1_ and *T*_2_ of each surface. Thus, there are four relaxation energies in total. Finally, the surface energy is computed for each of the four surfaces using Equation (3) as:

(3) $$E_{surf}=E_{Cl}^{\left(111\right),\;\left(100\right)}+\Delta E_{surf}^{rel\;\left(T_{1},\;T_{2}\right)}$$

The results for the four terminations are collected in Table 1, which, in agreement with a previous report by Zhang et al. [68], shows clearly that the (111) surfaces are more stable than the (100) surfaces.

#### 2.1.2. Stability of Doped Surfaces

As the heavy metal Pb in FAPI is harmful to both humans and the environment, it is particularly important to explore high-efficiency perovskite materials without, or with little, lead present in the structure. Following the doping of halide perovskite solar cell materials with alkaline earth metals in previous studies [69,70,71], we have created stable doped structures through substitution by environmentally friendly elements (Be, Mg, and Ca) according to the Goldsmith tolerance factor [72], where the creation of stable structures requires the value of this factor to lie in the range of 0.8–1. We have created various doped FAPI surfaces with different percentages of Be, Mg, and Ca metals, according to Equation (4), and only the stable structures with tolerance factors in the approved range are collected in Table 2.

(4) $$t=\frac{r_{\mathrm{FA}}+r_{I}}{\sqrt{2}\left([{\mathrm{xr}}_{\mathrm{Pb}}+\left(1-x\right)r_{\mathrm{AE}}]+r_{I}\right)}\;$$

where r_FA,_ r_I_, r_Pb_ and r_AE_ represent the ionic radii of FA^+^, I^−^, Pb^2+^ ions and alkaline earth metals, respectively, and x and (1−x) represent the percentages of Pb and alkaline earth metal dopants. As the (100) and (111) surfaces shown in Figure 2 contain the same number of lead atoms in their PbI_6_ terminations (5 layers: 20 Pb) they can undergo a similar doping percentage, as can the PbI_5_ and PbI_3_ terminations (6 layers: 24 Pb).

By increasingly doping alkaline earth metals at the lead sites of these surfaces, the tolerance factors start to exceed the upper allotted limit (t > 1) and unstable structures are created. Keeping this limit in mind, we have created various doped surfaces, including 6 different structures for each of the (100)-PbI_6_ and (111)-PbI_6_ terminations doped by 1 atom of Be, 1 atom of Mg, and 1–4 atoms of Ca, and 8 different structures for each of the (100)-PbI_5_ and (111)-PbI_3_ terminations doped by 1 atom of Be, 1–2 atoms of Mg, and 1–5 atoms of Ca, thereby obtaining a total of 28 different structures. Figure S1 shows all the doped structures within the permissible percentages of Be, Mg, and Ca doping.

### 2.2. Electronic Properties

The electronic structure calculations in this study are based on the GGA-PBE technique. The results of experimental methods and other computational functionals to calculate the FAPI bulk band gap were collected in a table in previous work [73]. The collected results [61,74,75,76] clearly show that the PBE functional is highly suitable for this work. Here, we discuss the electronic properties of the bulk material, the pristine (100) and (111) surfaces, and the Be-, Mg- and Ca-doped FAPI surfaces in the allowed percentages (0–0.2%). We have computed the total density of states (TDOS) and projected density of states (PDOS) on the Pb and I atoms of the bulk and surfaces of the FAPI perovskites. The energy states of the elements in FA (C, N, and H) are mainly distributed in the energy level below −5 eV, showing the weak interaction between the FA^+^ and Pb^2+^ and I^−^ [63].

#### 2.2.1. Electronic Properties of Bulk and Plain Surfaces

**Figure 3:**
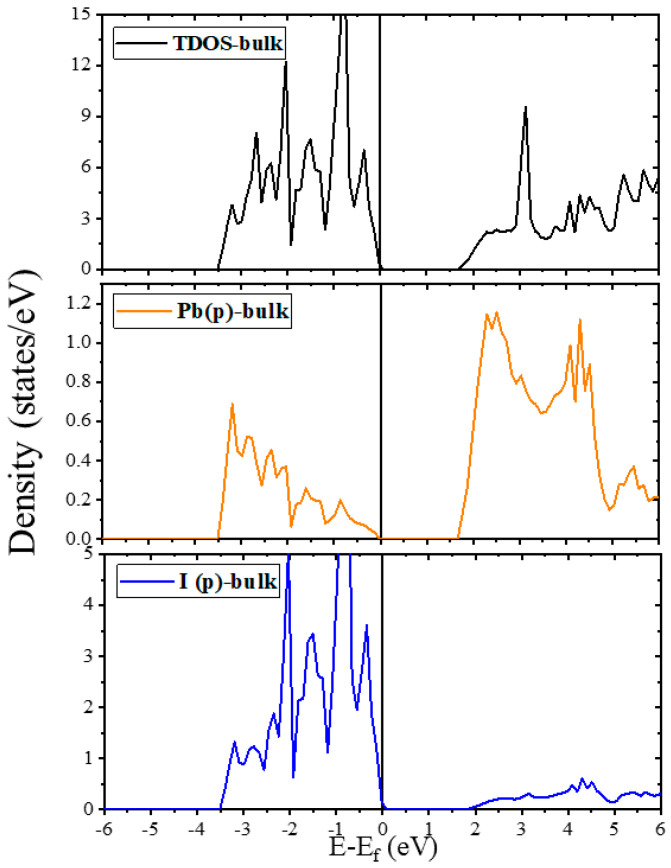
The total and partial density of states of FAPI bulk. The Fermi level is set at zero eV.

**Figure 4:**
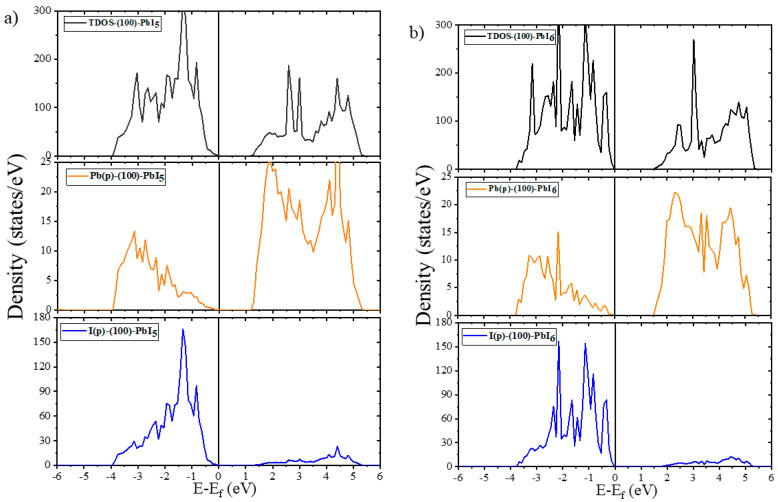

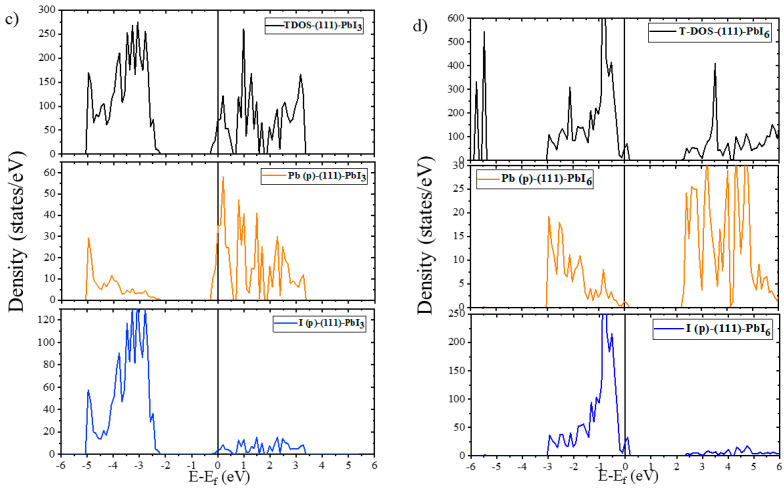
The total and partial density of states of FAPI surfaces (**a**) (100)-PbI_5_, (**b**) (100)-PbI_6_, (**c**) (111)-PbI_3_ and (**d**) (111)-PbI_6_. The Fermi level is set at zero eV.

The computed TDOS and PDOS spectra of the bulk and pristine surfaces are illustrated in Figure 3 and Figure 4. We have also compiled Table 3 for a better comparison of the structures and the exact location of the band gaps, valence and conduction bands (VB and CB) of both the plain and doped surfaces with the available data from the literature [61,74,75].

As shown in Figure 3, the calculated TDOS and PDOS spectra of the FAPI bulk exhibit a bandgap of 1.689 eV with the main contributions of the Pb-p and I-p orbitals in the CB and VB, respectively. The placement of the Fermi level at the top of the VB of the FAPI bulk indicates the properties of p-type semiconductors. The bandgap of the (100) surfaces are 1.309 and 1.623 eV for the PbI_5_ and PbI_6_ terminations, respectively, making them suitable for photovoltaic applications. The Fermi level for the (100) surfaces, as for the bulk, just catches the top of the VB, so these surfaces are also p-type semiconductors. According to the DOS diagrams of the (100) surfaces in Figure 4a,b, it can be seen that although the VB of both terminations coincide, the bandgap of the (100)-PbI_5_ termination is about 0.3 eV smaller than that of (100)-PbI_6_, due to the shift of the CB to higher energy areas in the (100) surface with PbI_6_ termination. A different trend is seen in the (111) surfaces, indicating that while the TDOS graph of the (111)-PbI_3_ surface shows interaction of the electronic states of the Pb-p orbitals with the Fermi level, in the (111)-PbI_6_ structure, the electronic states of the I-p orbitals intersect with the Fermi level due to the peak of those orbitals. These results indicate a zero bandgap and metal-like characteristics for the FAPI (111) surfaces.

#### 2.2.2. Electronic Properties of Doped Surfaces

In the next step, in order to understand the effect of dopants on the electronic properties of the surfaces, we have performed the related DOS calculations for all the alkaline earth metal-doped surfaces, listed in Table 3 and shown in Figures S2 and S3 for the (100) and (111) surfaces, respectively.

As shown in Figure S2 and Table 3, at the (100)-PbI_5_ surface, doping of all three alkali metals in all percentages slightly increased the bandgap. At the similarly structured (100)-PbI_6_ surface, doping of Ca and particularly Mg also slightly increased the bandgap, but Be-doping significantly reduced it. As shown in Figure S3, at the (111)-PbI_3_ surface, Be-doping and 0.08% of Mg-doping (FAPb_0.96_Be_0.04_I_3_ and FAPb_0.92_Mg_0.08_I_3_) have little effect, while 0.04% of Mg-doping (FAPb_0.96_Mg_0.04_I_3_) moves the electronic states of the I-p orbitals to the higher energy areas by 0.07 eV. Likewise, all percentages of Ca-doping slightly decreased the distance between the electronic states of the Pb-p and I-p orbitals, mainly by moving these orbitals to lower and higher energies, respectively, while 0.16% Ca-doping (FAPb_0.84_Ca_0.16_I_3_) led to a shift of the I-p orbital electronic states to lower energies by 0.09 eV. Finally, at the (111)-PbI_6_ surface, Be- and Mg-doping moved the electronic states to higher and lower energy areas, respectively, but in contrast to (111)-PbI_3_, Ca-doping increased the distance between the electronic states of the Pb-p and I-p orbitals, mainly by moving these orbitals to higher and lower energies, respectively, in particular in the FAPb_0.9_Ca_0.1_I_3_ structure.

In general, it can be said that on all surfaces, Ca-doping increases the bandgap or the distance between the electronic states of the Pb-p and I-p orbitals, except for (111)-PbI_3_ which is the only surface where the Pb-p orbitals intersect the Fermi level. The impact of Be- and Mg-doping in each termination varies depending on the doping-percentages, but as a whole, we observe that Be-doping has very little effect on the (111) surfaces, and different effects in decreasing and increasing the bandgaps of the (100)-PbI_6_ and (100)-PbI_5_ terminations, respectively. In the case of magnesium, doping increases the bandgap of the (100) surfaces by 0.1 eV, whereas the distance between the electronic states of the Pb-p and I-p orbitals in the (111) surfaces increases, except for the FAPb_0.96_Mg_0.04_I_3_ structure which does not change much.

### 2.3. Optical Properties of Bulk, Plain and Doped Surfaces

In this section, we discuss the photon energy-dependent optical properties of the pristine bulk and the two plain surfaces, in addition to the Be-, Mg- and Ca-doped surfaces of the FAPI perovskite.

#### 2.3.1. Dielectric Functions

The optical properties of a material are generally described by the dielectric function, as a function of photon energy, representing the linear response of any material to an external electromagnetic field, as defined by Equation (5):

(5) $$\varepsilon \left(\omega \right)=\varepsilon_{1}\left(\omega \right)+I\varepsilon_{2}\left(\omega \right)$$

where *ε*_1_(*ω*) and *ε*_2_(*ω*) represent the real and imaginary parts of the dielectric function *ε*(*ω*), respectively. *ε*_2_(*ω*) is computed using the following relationship [78]:

(6) $$Im(\varepsilon \left(\omega )\right)=i\varepsilon_{2}\left(\omega \right)=\left(\frac{e^{2}\hbar^{2}}{\pi m^{2}\omega^{2}}\right)\sum {\left|\langle \Psi_{C}\left|e_{j}.\;P^{⃗}\;\right|\Psi_{\nu }\rangle \right|}^{2}\delta (E_{C}-E_{v}-\hbar \omega )\;$$

In Equation (6), *P⃗* indicates the momentum operator, *e* and *m* are the charge and mass of a bare electron, respectively, *ê_j_* denotes the unit vector designating the direction of the external electromagnetic field of energy *ℏω*, and *E_v_* and *E_c_* are the related valence and conduction energies. It characterizes the optical absorption in the semiconductor, which is indicated by the inter-band transitions.

**Figure 5:**
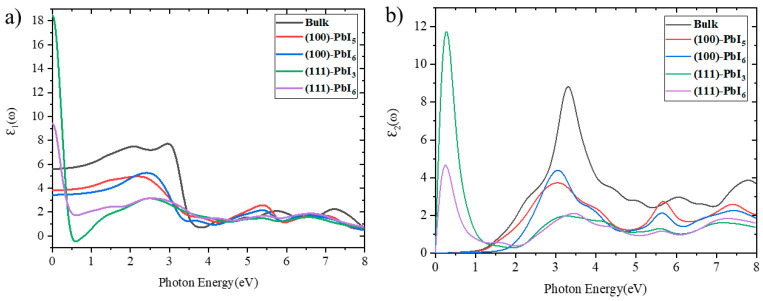
The (**a**) real and (**b**) imaginary parts of the dielectric function for FAPI bulk and (100) and (111) plain surfaces.

The real part of the complex dielectric function, *ε*_1_(*ω*), describes the dispersion of electromagnetic energy after the penetration into the medium, and is calculated from *ε*_2_(*ω*), using the Kramers–Kronig relations [79].

(7) $$Re\left(\varepsilon \left(\omega \right)\right)=\varepsilon_{1}\left(\omega \right)=1+\frac{2}{\pi }P\overset{\infty }{\underset{0}{\int }}\frac{{\omega \prime \varepsilon }_{2}\left(\omega \prime \right)}{\omega^{\prime 2}-\omega^{2}}d\omega \prime$$

where P is the Cauchy principal value of the integral. Figure 5 depicts the computed real, *ε*_1_(*ω*), and imaginary, *ε*_2_(*ω*), parts of the dielectric function of the FAPI bulk and plain surfaces. As can be seen in Figure 5a, the primary characteristics of the *ε*_1_(*ω*) spectrum of the bulk are three spectral peaks with magnitudes of 7.71, 2.07, and 2.24 at photon energies of around 2.95, 5.73 and 7.23 eV, respectively, whereas these peaks move to lower photon energy regions and the magnitude of the first peak is reduced by about 2.5 and 4 units in the (100) and (111) surfaces, respectively. Interestingly, the magnitude of the second peak of the (100) surfaces is larger than that of the bulk, with a rapid drop reaching a minimum at about 3.83 eV. As expected, the real part of the dielectric function converges to a constant at higher photon energies. The computed static dielectric constants, *ε*_1_(0), which designates the dielectric response of a material to a static electric field, are calculated at around 5.61, 3.82, 3.43 for the bulk, (100)-PbI_5_ and (100)-PbI_6_ structures, respectively, and, surprisingly, at around 18.5 and 9.41 for the (111)-PbI_3_ and (111)-PbI_6_ surfaces, respectively. In the (111) surfaces, one can see a steep start of the spectrum, while the bulk and (100) surfaces show a gradual upward trend. The similarity of the spectra for the bulk and (100) surfaces indicates a similarity in optical properties.

According to Figure 5b, the *ε*_2_(*ω*) spectra of the bulk and (100) surfaces have a zero value until the absorption commences after the photon energy reaches the band gap energy. This provides the threshold for the direct optical transition between the highest VB and the lowest CB. Based on the imaginary term of the dielectric function *ε*_2_(*ω*), the optical absorption edge for these structures starts at about 1 eV. This property is different for the (111) surfaces, where the imaginary part of the dielectric function of the (111) surfaces starts from a zero value of the photon energy, but immediately increases to the higher *ε*_2_(*ω*) and creates an infrared peak. Regardless of the initial peaks of the (111) surfaces in low-energy areas, the *ε*_2_(*ω*) spectra illustrate relatively sharp essential peaks around 3.3 eV for the bulk and 3.06 eV for the (100) surfaces. Our results corroborate well previous experimental and theoretical results, cited in Refs [80,81].

The magnitude of *ε*_1_(0), as shown in Figure S4, decreases for all alkaline earth metal-doped structures compared to the plain surfaces, where the largest decrease was observed for the (111) surface with the highest percentage of calcium doping (FAPb_0.8_Ca_0.2_I_3_). The main visible *ε*_1_(*ω*)-peak of the (111) surfaces does not change much with doping, but the doping effect is seen in a reduction of the height of this peak on the (100)-PbI_6_ surface. On the (100)-PbI_5_ surface, a different trend is observed, where Be-doping (FAPb_0.96_Be_0.04_I_3_) and high percentages of Ca-doping (FAPb_0.8_Ca_0.2_I_3_, FAPb_0.84_Ca_0.16_I_3_ and FAPb_0.88_Ca_0.12_I_3_) slightly reduce the height of the main peak, whereas replacement by Mg (FAPb_0.96_Mg_0.04_I_3_ and FAPb_0.92_Mg_0.08_I_3_) and low percentages of Ca (FAPb_0.92_Ca_0.08_I_3_ and FAPb_0.96_Ca_0.04_I_3_) cause a slight increase in the magnitude of *ε*_1_(*ω*), at 2.19 eV. It is worth mentioning that in the region around 2.8 to 4.6 eV, the curves of all doped (100) surfaces are above those of the plain surfaces.

The magnitude of *ε*_2_(*ω*) in the visible area, as shown in Figure S5, decreases particularly for high percentages of Ca (FAPb_0.8_Ca_0.2_I_3_) in the doped (100) surfaces of both terminations. The *ε*_2_(*ω*) of (111)-PbI_3_ does not show any significant changes after doping in all areas, except for the initial peak in the infrared region, whose height has reduced significantly in the FAPb_0.8_Ca_0.2_I_3_ structure. Substitution of alkaline earth metals at the (111)-PbI_6_ surface results in a very subtle decrease in the magnitude of *ε*_2_(*ω*) in most areas. It is worth noting that at around 4.3 to 5.3 eV, the curves of all (100) doped surfaces are higher than for both plain surfaces.

Once both the real and imaginary terms of the photon energy-dependent dielectric function are provided, valuable optical characteristics can be established, as described in the following sub-sections.

#### 2.3.2. Refractive Index and Extinction Coefficient

The identification of the refractive index is essential for optoelectronic devices, where it characterizes the measure of the material’s transparency to photons. The refractive index is connected to the degree that the speed of light is diminished through a material, compared to the speed of light in a vacuum. The complex refractive index of a material, $\tilde{n}$(*ω*), is denoted by the following formula:

(8) $$\tilde{n}\left(\omega \right)=n\left(\omega \right)+ik\left(\omega \right)=\varepsilon^{\frac{1}{2}}={\left(\varepsilon_{1}+i\varepsilon_{2}\right)}^{\frac{1}{2}}\;$$

where *n*(*ω*) describes the real component of the refractive index, while *k*(*ω*) represents the imaginary component or the extinction coefficient [78]. They are described as:

(9) $$n\left(\omega \right)={\left[\frac{\varepsilon_{1}\left(\omega \right)}{2}+\sqrt{\frac{\varepsilon_{1}^{2}\left(\omega \right)+\varepsilon_{2}^{2}\left(\omega \right)}{2}}\right]}^{\frac{1}{2}}\;$$

(10) $$k\left(\omega \right)={\left[\frac{-\varepsilon_{1}\left(\omega \right)}{2}+\sqrt{\frac{\varepsilon_{1}^{2}\left(\omega \right)+\varepsilon_{2}^{2}\left(\omega \right)}{2}}\right]}^{\frac{1}{2}\;}\;$$

**Figure 6:**
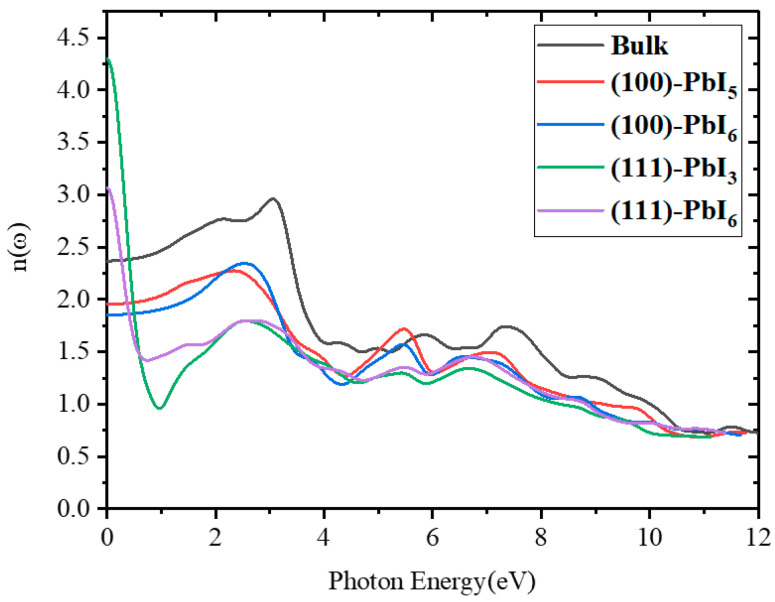
The refractive index spectra for FAPI bulk and plain surfaces.

To measure the transparency of FAPI systems to incident light, the theoretical refractive index values were computed by means of Equation (9) and portrayed in Figure 6. As is apparent, *n*(*ω*) is not altered significantly for photon energies upward of the band gap energy, whereas an important variation is discerned at the photon energies in the visible regime which both suggest optically stable materials. Similar to the underlying gap and the static dielectric constant *ε*_1_(0), the static refractive index *n*(0) is also a valuable physical quantity for semiconductors. The calculated values of *n*(0) are 2.37, 1.95, 1.85, 3.05 and 4.22 for the bulk material and the (100)-PbI_5_, (100)-PbI_6_, (111)-PbI_6_ and (111)-PbI_3_ surfaces, respectively. The main peaks in the refractive index spectra of the FAPI structures are between 2.4 and 3.1 eV, which agrees well with previous investigations that have reported refractive indices of FAPI ranging between 2.2 and 2.7 [80,82,83]. It has been suggested previously for MAPI films that deviations in the refractive index stem from the differences in layer thickness, morphology, chemical composition and material anisotropy [7,84,85].

According to Figure S6, the values of n(0) of all doped structures have decreased compared to those of the plain structures, which can be seen particularly clearly in the (111)-PbI_3_ surface with FAPb_0.8_Ca_0.2_I_3_ structure. The visible peak of the refractive index curves of the (111) doped surfaces does not change significantly compared to the plain surface, but the doping of alkaline earth metals at the (100) surfaces shows two different effects; below around 3 eV, the peaks have decreased, but the opposite occurs beyond this energy until the end of the visible area, and an increasing effect on *n*(*ω*) is observed.

**Figure 7:**
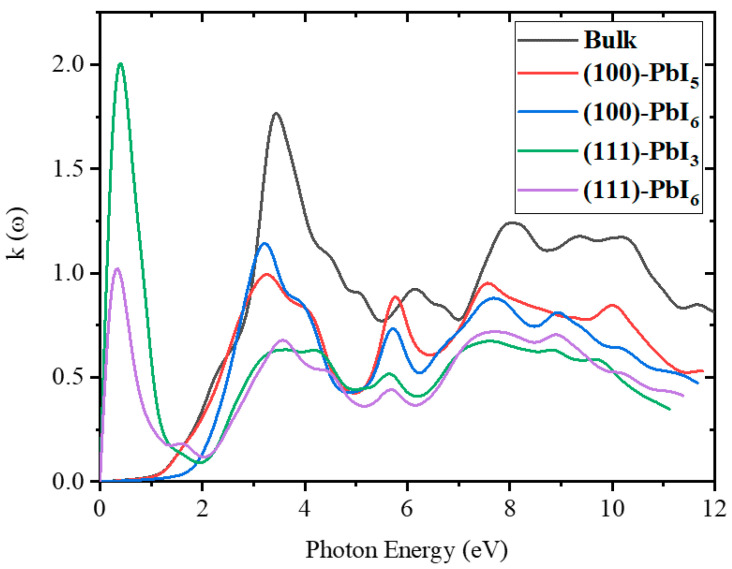
The extinction coefficient for FAPI bulk and plain surfaces.

According to Figure 7, the pristine FAPI bulk shows extinction coefficient peaks at 3.52, 6.25, and 8.15 eV with k values of 1.74, 0.9, and 1.24, respectively. The first peaks of the (111) surfaces are at low photon energies, while beyond 2 eV all structures follow the same trend as the bulk. The magnitude of k for the (100) surfaces is bigger than those of the (111) surfaces, but smaller than the bulk. These data are extremely important for the design of the optical features of perovskite-based solar cells.

As shown in Figure S7, at 4.4 to 5.4 eV, the extinction coefficients of all doped (100) structures are higher than those of the plain surfaces. In the visible area, the main peaks of the doped (100) and (111)-PbI_6_ surfaces have decreased compared to those of the plain surfaces, with the largest decrease for the FAPb_0.8_Ca_0.2_I_3_ structures. The doped (111)-PbI_3_ graph does not show any significant changes compared to that of the plain surface in the visible region, while the infrared peak of the (111) surfaces decreases in all doped structures.

#### 2.3.3. Reflectivity

It is important in photovoltaic devices to investigate the surface reflection properties of the relevant materials, where normally the reflectivity is the most cited optical measurement of a material. The reflectivity or the reflection coefficient of the material describes the reflection at the surface and using Equations (9) and (10), the reflectivity *R*(*ω*) can be computed according to Equation (11):

(11) $$R\left(\omega \right)=\frac{{\left(n-1\right)}^{2}+k^{2}}{{\left(n+1\right)}^{2}+k^{2}}={\left|\frac{\sqrt{\varepsilon }-1}{\sqrt{\varepsilon +1}}\right|}^{2}$$

**Figure 8:**
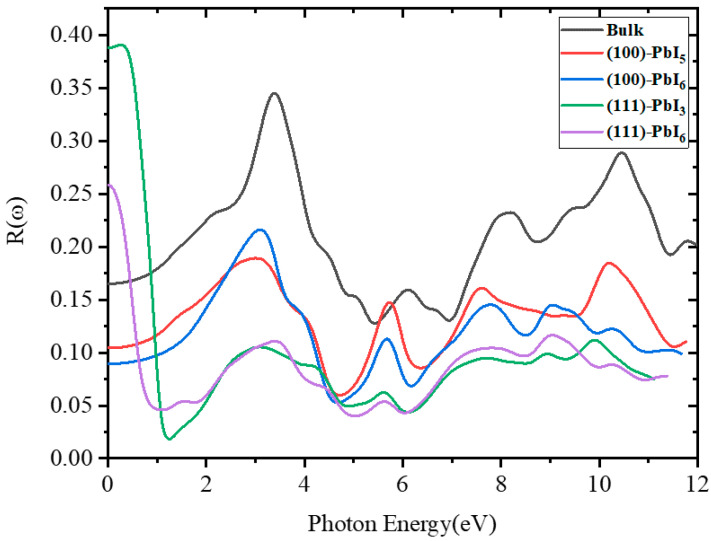
The reflectivity spectra for FAPI bulk and plain surfaces.

The computed reflectivity spectra of the FAPI bulk and plain surfaces are shown in Figure 8, where prominent peaks are detected at around 3.48 eV for the bulk and (111)-PbI_6_ structure and at 3.1 eV for the other surfaces, with significant reflectivity extending up to about 10 eV. When the absorption is intense, the reflectivity is insignificant and the material effectively reflects light in some regimes, but it cannot absorb light in the same area. Interestingly, the *R*(0) of the bulk is about 16% at zero photon energy which confirms the intense and valuable absorptivity of this material. This value decreases for the (100) surfaces and increases for the (111) surfaces, especially the (111)-PbI_3_ structure which reached over 38%.

According to Figure S8, the value of *R*(0) in all doped structures has decreased compared to the plain structures, especially in the (111)-PbI_3_ surface with the FAPb_0.8_Ca_0.2_I_3_ structure. It is also observed that the spectra of all the doped structures are higher than those of the plain structures in the photon energy range of 4.3 to 5.3 eV. The magnitude of the visible peaks in all the doped (100) and (111)-PbI_6_ structures has decreased, while Be and Mg doping had little effect on the main peak of the (111)-PbI_3_ structure, and only a very small increase of *R*(*ω*) is observed in the FAPb_0.96_Ca_0.04_I_3_, FAPb_0.92_Ca_0.08_I_3_ and FAPb_0.88_Ca_0.12_I_3_ structures.

#### 2.3.4. Energy Loss Spectrum

Electron energy loss spectroscopy (EELS) characterizes information on the elastically scattered and non-scattered electrons, as well as the atomic number of any atoms irradiated by the electron beam [7,84]. The energy loss function, *L*(*ω*), is described by the following relationship:

(12) L(ω)=−Im(1ε(ω))=ε2(ω)ε12(ω)+ε22(ω) 

**Figure 9:**
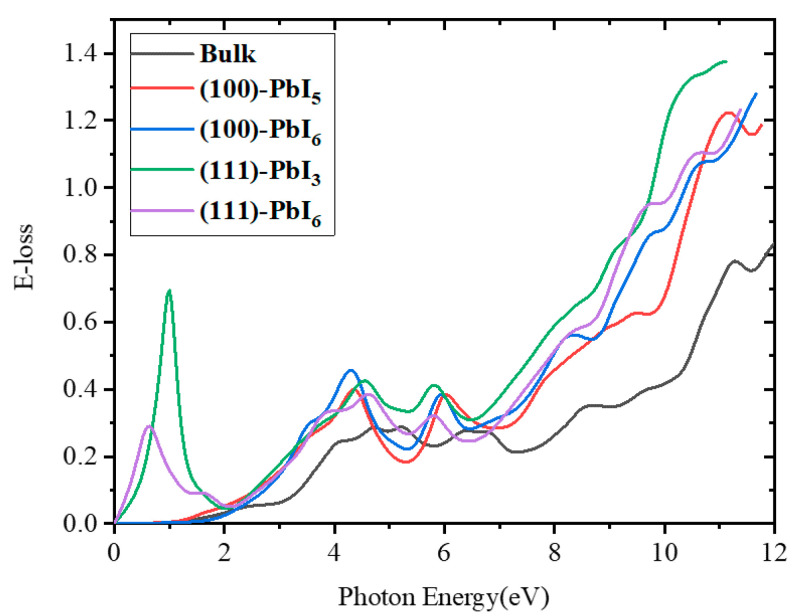
The electron energy loss function versus photon energy for FAPI bulk and plain surfaces.

Electron energy loss functions for the FAPI bulk and (100) and (111) surfaces are illustrated in Figure 9. There is no energy loss in the case of photons with energies smaller than the band gap of the bulk and (100) surfaces, in contrast with the (111) surfaces, indicating no scattering by the bulk and (100) surfaces. For the intermediate energy span over 11 eV, inelastic scattering was detected and thus the maximum value of energy loss is attainable for all structures. A substantial intensity peak is noted at about 11.3 eV for the FAPI systems.

As shown in Figure S9, the peaks at the end of the visible area (around 4.4 eV) shrink for all the doped surfaces compared with those for the plain surfaces, except for the FAPb_0.96_Be_0.04_I_3_, FAPb_0.96_Mg_0.04_I_3_, and FAPb_0.96_Ca_0.04_I_3_ structures of the (111)-PbI_3_ surface. The infrared peak from the (111)-PbI_6_ surface does not change with doping, but at (111)-PbI_3_ only doping by Mg has no effect. In other words, doping with Be and all percentages of Ca, except 16% (FAPb_0.84_Ca_0.16_I_3_), reduces the height of this peak. In the range of 4.6 to 5.9 eV, the curves of the doped (100) structures are higher than those of the plain surfaces.

#### 2.3.5. Absorption Coefficient

The absorption coefficient, *α*(*ω*), characterizes the amount of light absorbed by a material. The absorption coefficient is a function of the photon energy; where the photon energy does not exceed the band-gap, electron excitation will not occur and the crystal is transparent. Using parts of the dielectric function or the extinction coefficient, *K*(*ω*) (Equation (10)), one can obtain the absorption coefficient from the following expression:

(13) $$\alpha \left(\omega \right)=2\omega k\left(\omega \right)=2\omega {\left[\frac{-\varepsilon_{1}\left(\omega \right)}{2}+\sqrt{\frac{\varepsilon_{1}^{2}\left(\omega \right)+\varepsilon_{2}^{2}\left(\omega \right)}{2}}\right]}^{\frac{1}{2}}$$

**Figure 10:**
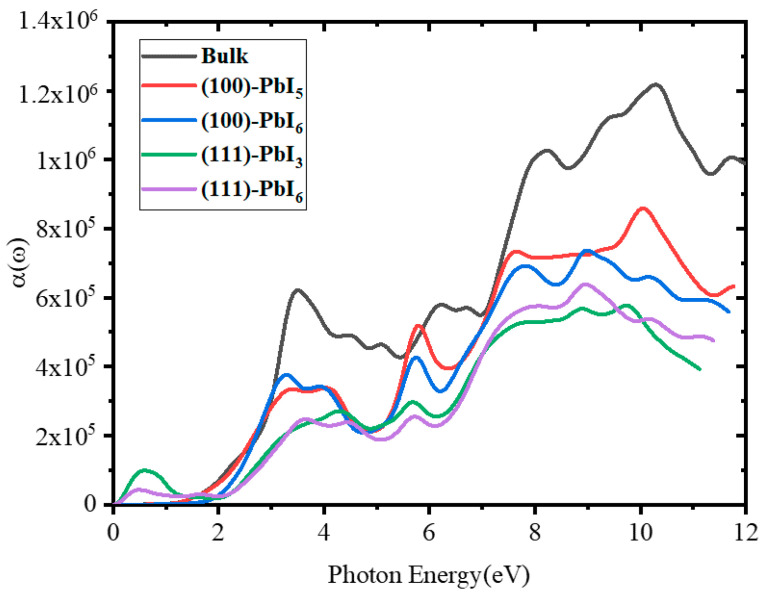
The absorption coefficient spectra for FAPI bulk and plain surfaces.

Figure 10 shows the absorption coefficient of the FAPI bulk and plain surfaces as a function of the photon energy. Three clear peaks can be distinguished in the spectrum of the bulk material at approximately 3.48, 8.24 and 10.4 eV, whereas the absorption is insignificant in the lower energy region from 0 to 1.41 eV, indicating that the material is transparent in the partially ultra-violet to the visible light window. The surface spectra show a similar trend to that of the bulk, although, surprisingly, the (100) surfaces show noticeable peaks at 5.8 eV in addition to the peaks mentioned for the bulk, with the (100)-PbI_6_ surface producing even higher absorption coefficients than the bulk at this point. We observed that overall the absorption coefficient of the (100) surfaces is higher than that of the (111) surfaces. At lower energies up to 3 eV, the spectra of the bulk and (100)-PbI_5_ surface are equal, while beyond this energy, at around 3.3 eV, the (100)-PbI_6_ surface shows more absorption than the other surface. It is obvious that the maximum absorption coefficient arises at an energy of 10.4 eV, where the peak of the (100)-PbI_5_ surface is higher than those of the other surfaces.

**Figure 11:**
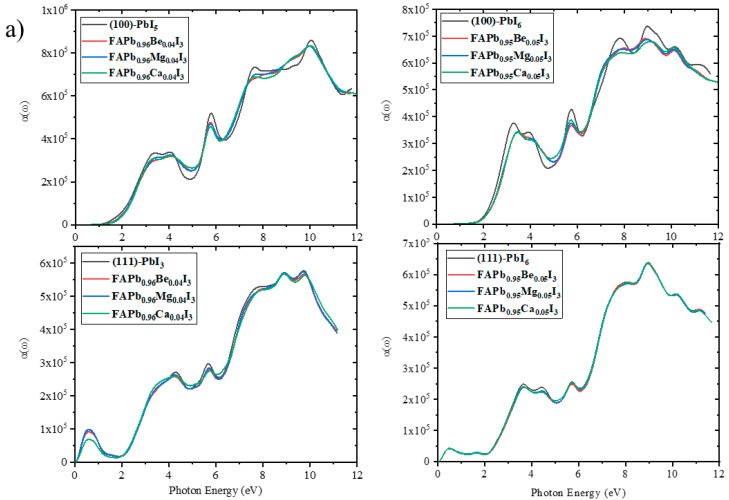

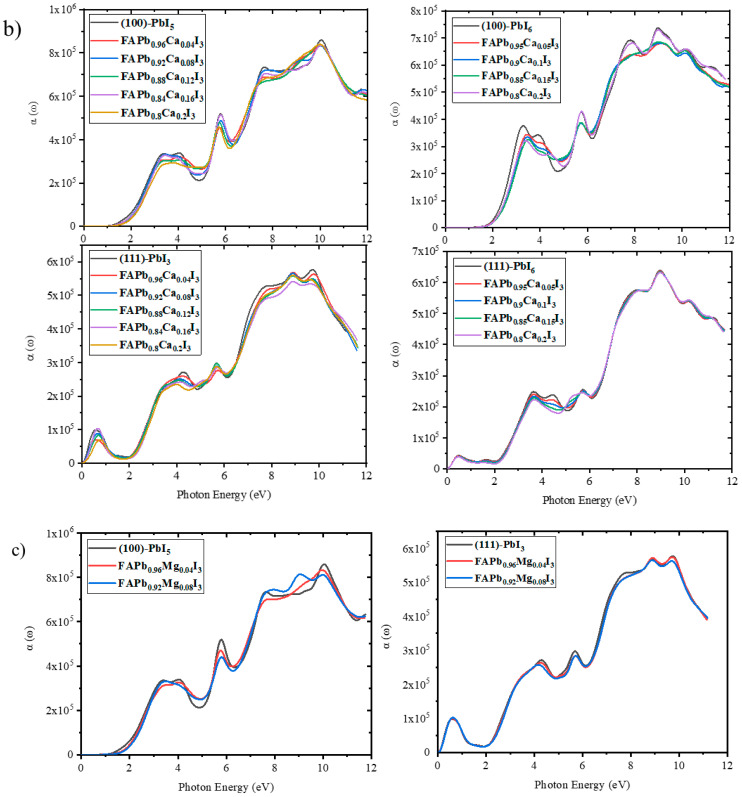
The absorption coefficient spectra for (100) and (111) surfaces with doping of (**a**) 1 atom of Be, Mg, and Ca in all terminations, (**b**) 1-4 atoms of Ca in both PbI_6_ terminations and 1-5 atoms of Ca in PbI_5_ and PbI_3_ terminations, and (**c**) 1-2 atoms of Mg in PbI_3_ and PbI_5_ terminations.

For the doped surfaces, as shown in Figure 11, the magnitude of the first peak in the absorption coefficient spectra has decreased for all structures, especially in the presence of high percentages of Ca (FAPb_0.8_Ca_0.2_I_3_). However, a slight increase of this peak can be observed in the (111)-PbI_3_ structure, especially in the presence of low percentages of Ca (FAPb_0.96_Ca_0.04_I_3_). In addition, the infrared peak for this surface shrinks for all doped structures except for the FAPb_0.84_Ca_0.16_I_3_ structure. At 4.5 to 5.3 eV, the absorption coefficients of all the (100) doped structures are higher than those of the plain surfaces.

### 2.4. Light Harvesting Efficiency (LHE)

From the results of the previous section and the magnitudes of the absorption coefficients, one can calculate the absorbance quantity by the following equation:

(14) $$\alpha =2.303\frac{A}{k}\;$$

where α, A and k represent the absorption coefficient, absorbance and sample thickness, respectively. Using absorbance, A, in Equation (15) [86], we obtain the amount of light harvested by the structures:

(15) $$\mathrm{LHE}=1-10^{-A}\;$$

**Figure 12:**
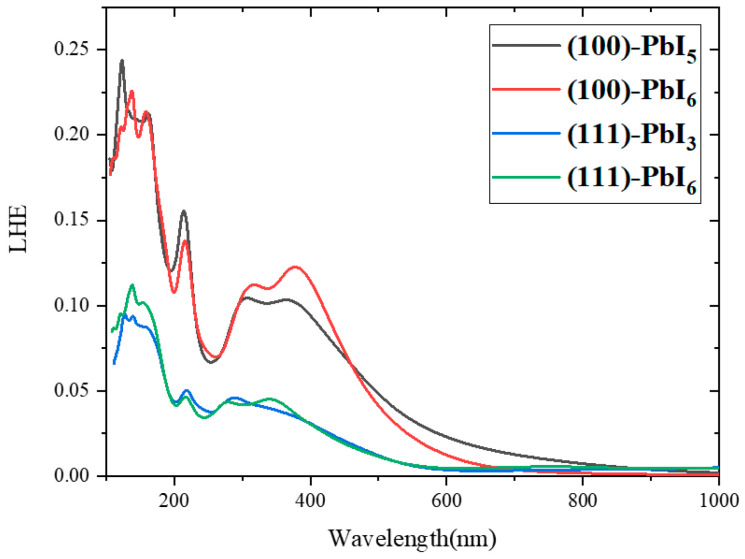
The light harvesting efficiency spectra for FAPI bulk and plain surfaces.

**Figure 13:**
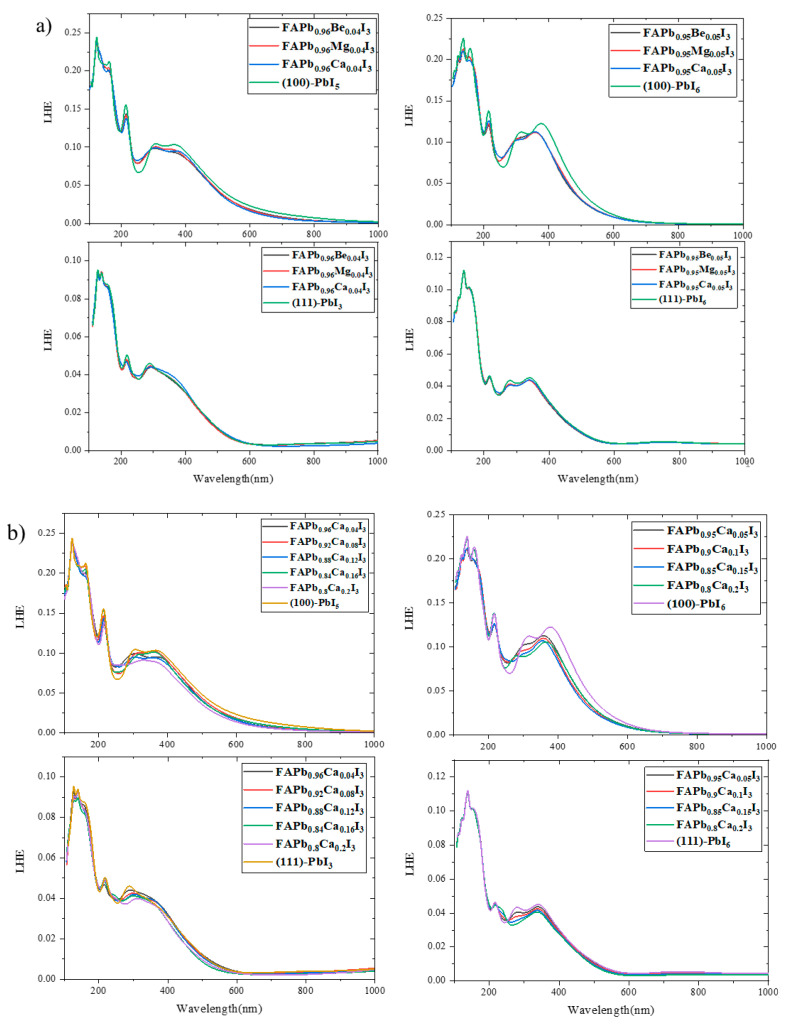

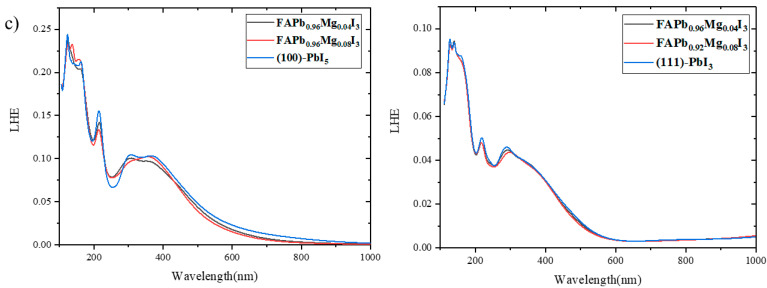
The light harvesting efficiency spectra for (100) and (111) surfaces with doping of (**a**) 1 atom of Be, Mg, and Ca in all terminations, (**b**) 1-4 atoms of Ca in both PbI_6_ terminations and 1-5 atoms of Ca in PbI_5_ and PbI_3_ terminations, and (**c**) 1-2 atoms of Mg in PbI_3_ and PbI_5_ terminations.

In Figure 12 and Figure 13, the graphs show the ability of the surfaces to absorb sunlight versus the photon energy. Using these graphs, it is easy to compare the amount of LHE between the plain and doped surfaces. As can be seen in Figure 12, the light harvested by the (100) surfaces in both the UV and visible areas is more than that harvested by the (111) surfaces. In the initial wavelengths of the visible regions (380 nm), it is observed that the (100)-PbI_6_ surface has the highest light-harvesting efficiency, and the better optical properties compared to the other three surfaces. Additionally, its bandgap differs least from the bulk, which is the optimal gap for photovoltaic applications. In the lower energy visible area after around 460 nm, the spectrum of the other termination of the (100) surface, (100)-PbI_5_, is higher than the others and the (100) surfaces therefore perform better in the visible area than the (111) surfaces.

A comparison of the doped surfaces in Figure 13 with the plain surfaces shows that in all surfaces doping by Be, Mg and Ca in various amounts either reduces LHE (on the (100) surface) or has no effect (e.g., (111)-PbI_6_). The only small positive effect of doping on LHE occurred in the initial visible area for the relatively low percentage Ca-doped (111)-PbI_3_ surface with FAPb_0.96_Ca_0.04_I_3_, FAPb_0.92_Ca_0.08_I_3_, and FAPb_0.88_Ca_0.12_I_3_ structures.

## 3. Computational Methods

We have employed calculations based on the density functional theory (DFT) as implemented in the Vienna ab initio simulation package [87] (VASP 5.4.4). The exchange-correlation functional developed by Perdew, Burke and Ernzerhof (PBE) and the Generalized Gradient Approximation (GGA) [88] were employed with the dispersion correction using Grimme’s [89] DFT-D3 scheme to obtain the structural and electronic properties. The Projector-Augmented-Wave (PAW) pseudopotentials were utilized for the geometry optimization, wherein all the structures and atomic positions were fully relaxed and optimized using the conjugate gradient algorithm without any symmetric constraint. The convergence criteria were set so that the total energy variation per atom is less than 10^−5^ eV and a sufficiently high kinetic energy cutoff of 450 eV was chosen for the plane wave expansion. Based on convergence assessments 2 × 2 × 2 and 5 × 5 × 1 Monkhorst-Pack k-point meshes were chosen for the Brillouin-zone sampling of bulk and surfaces, respectively, of the perovskite structures (K grid point considered 1 for the z-direction of the surface).

The surfaces were modelled by a periodic slab separated by at least 20 Å of vacuum, including 20 and 24 units of FA, 5 and 6 layers of Pb, and various numbers of I atoms for the ((111)-PbI_6_, (100)-PbI_6_) and ((111)-PbI_3_, (100)-PbI_5_) surfaces, respectively. When creating the surfaces from the bulk material, using Materials Studio, two independent terminations were identified for each of the two surface orientations, and we have modelled both terminations for each surface to compare and contrast the effect of the different surface configurations on the materials properties. The convergence of the number of Pb layers and vacuum space was confirmed by the study of Haruyama and coworkers [90].

## 4. Conclusions

The results from this study show that both plain and doped FAPI (100) and (111) surfaces are stable, and that doping by various alkaline earth metals in different PbI_5_, PbI_3_ and PbI_6_ terminations of these surfaces have important effects on the electronic and optical properties. We conclude that although the (111) surface has better stability than the (100) surface, it does not exhibit suitable properties for optical applications, as the Fermi level crosses the electronic states, indicating metallic properties.

Our calculations of the electronic properties confirm that the main contribution of the Pb-p orbitals is in the conduction band and the I-p orbitals in the valence band. Different terminations of each surface affect their electronic properties. In the (111) surfaces, the PbI_3_ termination shows interaction of the electronic states of the Pb-p orbitals with the Fermi level, while in PbI_6_ termination the I-p orbitals intersect the Fermi level. Additionally, the PbI_6_ termination increases the distance between CB and VB by about 0.135 eV compared to PbI_3_. In the (100) surfaces, shifting the CB of the PbI_6_ termination to higher energy areas again leads to an increase of about 0.3 eV in its bandgap compared to the PbI_5_ termination. The (100) surfaces exhibit a suitable bandgap of around 1.309 and 1.623 eV for the PbI_5_ and PbI_6_ terminations, respectively, which make them promising candidates for electronic applications.

The density of states diagrams of the doped (100) structures revealed an exciting outcome for all alkaline earth metal dopants, i.e., a critical 0.315 eV decrease and 0.096 eV increase in the bandgap was observed in the FAPb_0.95_Be_0.05_I_3_ and FAPb_0.96_Be_0.04_I_3_ structures of the (100) surface with the PbI_6_ and PbI_5_ terminations, respectively, while Mg- and Ca-doped (100) structures showed increasing bandgaps, notably in the FAPb_0.96_Ca_0.04_I_3_ and FAPb_0.8_Ca_0.2_I_3_ structures of the (100)-PbI_5_ surface, with bandgaps of 1.459 and 1.468 eV, respectively. The distance between the electronic states of the Pb-p and I-p orbitals of the (111)-PbI_6_ surface in Be- and Mg-doped structures effectively does not change, but in all Ca-doped structures of this surface, the gap increased, particularly in the FAPb_0.9_Ca_0.1_I_3_ structure by about 0.14 eV. The distance between the electronic states of the Pb-p and I-p orbitals in the FAPb_0.96_Mg_0.04_I_3_ structure of the (111)-PbI_3_ surface showed a decrease of 0.095 eV, while there were no significant changes in the gap in the FAPb_0.92_Mg_0.08_I_3_ and FAPb_0.96_Be_0.04_I_3_ structures. In the case of the Ca-doped (111)-PbI_3_ structures, this distance reduced, except for the FAPb_0.84_Ca_0.16_I_3_ structure which showed an 0.075 eV gap increase.

The comparison between the absorption spectra of the different terminations of the plain (100) surface shows that the (100)-PbI_5_ has higher photovoltaic efficiency in all spectral regions, except in the range of 300 to 460 nm where the (100)-PbI_6_ surface shows more absorption. Comparison of the other optical properties of the (100) terminations do not reveal much difference. However, in the (111) surfaces, the (111)-PbI_3_ termination shows higher infrared peaks of the imaginary part of the dielectric function, differences in the energy loss spectrum, absorption and extinction coefficient, and also a higher starting point of the real part of the dielectric function, and differences in the reflectivity and refractive index, compared with those of the PbI_6_ termination.

All doped surfaces have the capacity to absorb more photons in the near UV region compared to their plain counterparts, seen predominantly in the doped (100) structures. Our results indicate that the FAPb_0.96_Ca_0.04_I_3_, FAPb_0.92_Ca_0.08_I_3_ and FAPb_0.88_Ca_0.12_I_3_ structures of the (111)-PbI_3_ surface have better optical properties in the initial visible areas, compared to the other doped (111)-PbI_3_ surfaces and even its plain structures. Thus, in order to improve the properties of this surface, which is especially stable, doping with 0.04, 0.08, and 0.12% of calcium in the lead site could be a good strategy.

In summary, the surfaces exhibit the following different properties, which affect the results and their potential efficacy as photovoltaic materials. Despite the higher stability of the (111) compared to the (100) surfaces, according to the DOS diagrams and the location of the Fermi level the (111) surfaces exhibit metallic behaviour. In contrast, the electronic properties of the (100) surfaces are more tunable and their band gaps are more suitable and very close to the appropriate level for photovoltaic applications. Interestingly, the optical properties and LHE calculations show that the (100) surfaces are much more like the FAPI bulk than the (111) planes. The plain (100) surfaces exhibit promising optical activity in the visible and UV windows and indicate a remarkably high extinction coefficient, light harvesting efficiency and better optimal absorption compared to the (111) surfaces, which indicate the superiority of the (100) surfaces for photovoltaic applications.

The calculations performed in this work have provided an in-depth overview of the electronic and optical properties of an important perovskite, which we consider will help to accelerate the development of stable and non-toxic perovskite solar cells by predicting suitable compositions and structures for further experimental validation.

## Figures and Tables

**Table 1:** Surface energy values of the (100) and (111) surfaces of FAPI perovskite with different terminations.

Surfaces	$E_{Slab}^{unrel}$ (eV)	$E_{slab}^{rel}$ (eV)	$E_{Cl}$ (eV/A°^2^)	$\Delta E_{Surf}^{rel}$ (eV/A°^2^)	$E_{surf}$ (eV/A°^2^)
(1 0 0)-PbI_5_	−1162.813	−1165.512	0.059	−0.009	0.051
(1 0 0)-PbI_6_	−1315.501	−1318.417	0.059	−0.009	0.050
(1 1 1)-PbI_3_	−1129.200	−1136.183	0.048	−0.011	0.037
(1 1 1)-PbI_6_	−1325.358	−1331.876	0.048	−0.010	0.038

**Table 2:** The calculated results of Goldschmidt tolerance factor (t) for surfaces with various percentages and number of alkaline earth metal-doping.

(100)-PbI_5_ and (111)-PbI_3_			(100)-PbI_6_ and (111)-PbI_6_		
Number-Percentage of the Doped Metals	Doped Surface	t	Number-Percentage of the Doped Metals	Doped Surface	t
1 atom-0.04Be	FAPb_0.96_Be_0.04_I_3_	0.996	1 atom-0.05Be	FAPb_0.95_Be_0.05_I_3_	0.997
1 atom-0.04Mg	FAPb_0.96_Mg_0.04_I_3_	0.992	1 atom-0.05Mg	FAPb_0.95_Mg_0.05_I_3_	0.993
2 atoms-0.08Mg	FAPb_0.92_Mg_0.08_I_3_	0.998			
1 atom-0.04Ca	FAPb_0.96_Ca_0.04_I_3_	0.989	1 atom-0.05Ca	FAPb_0.95_Ca_0.05_I_3_	0.989
2 atoms-0.08Ca	FAPb_0.92_Ca_0.08_I_3_	0.991	2 atoms-0.1Ca	FAPb_0.9_Ca_0.1_I_3_	0.992
3 atoms-0.12Ca	FAPb_0.88_Ca_0.12_I_3_	0.993	3 atoms-0.15Ca	FAPb_0.85_Ca_0.15_I_3_	0.995
4 atoms-0.16Ca	FAPb_0.84_Ca_0.16_I_3_	0.996	4 atoms-0.2Ca	FAPb_0.8_Ca_0.2_I_3_	0.998
5 atoms-0.2Ca	FAPb_0.8_Ca_0.2_I_3_	0.998			

**Table 3:** The band gaps, valence, and conduction bands of the bulk plain and Be, Mg, and Ca-doped (111) and (100) surfaces of FAPI perovskite.

Structure	Bandgap	Valence Band	Conduction Band
Bulk	1.689, 1.7 [76], 1.72 [61], 1.75 [77], 1.58 [75], 1.53 [74]	−0.026	1.663
(1 1 1)-PbI_3_	0	–2.284	−0.299
FAPb_0.96_Be_0.04_I_3_	0	−2.282	−0.293
FAPb_0.96_Mg_0.04_I_3_	0	−2.208	−0.318
FAPb_0.92_Mg_0.08_I_3_	0	−2.315	−0.323
FAPb_0.96_Ca_0.04_I_3_	0	−2.201	−0.305
FAPb_0.92_Ca_0.08_I_3_	0	−2.265	−0.297
FAPb_0.88_Ca_0.12_I_3_	0	−2.204	−0.334
FAPb_0.84_Ca_0.16_I_3_	0	−2.374	−0.310
FAPb_0.8_Ca_0.2_I_3_	0	−2.275	−0.319
(1 1 1)-PbI_6_	0	0.088	2.208
FAPb_0.95_Be_0.05_I_3_	0	0.093	2.210
FAPb_0.95_Mg_0.05_I_3_	0	0.074	2.192
FAPb_0.95_Ca_0.05_I_3_	0	0.023	2.187
FAPb_0.9_Ca_0.1_I_3_	0	0.017	2.281
FAPb_0.85_Ca_0.15_I_3_	0	0.052	2.214
FAPb_0.8_Ca_0.2_I_3_	0	0.057	2.216
(1 0 0)-PbI_5_	1.309	−0.126	1.183
FAPb_0.96_Be_0.04_I_3_	1.405	−0.025	1.380
FAPb_0.96_Mg_0.04_I_3_	1.409	−0.126	1.283
FAPb_0.92_Mg_0.08_I_3_	1.408	−0.050	1.358
FAPb_0.96_Ca_0.04_I_3_	1.459	−0.130	1.329
FAPb_0.92_Ca_0.08_I_3_	1.350	−0.026	1.324
FAPb_0.88_Ca_0.12_I_3_	1.365	−0.053	1.312
FAPb_0.84_Ca_0.16_I_3_	1.355	−0.130	1.225
FAPb_0.8_Ca0.2I_3_	1.468	−0.052	1.416
(1 0 0)-PbI_6_	1.623	−0.127	1.496
FAPb_0.95_Be_0.05_I_3_	1.308	−0.126	1.182
FAPb_0.95_Mg_0.05_I_3_	1.714	−0.126	1.588
FAPb_0.95_Ca_0.05_I_3_	1.659	−0.052	1.607
FAPb_0.9_Ca_0.1_I_3_	1.657	−0.129	1.528
FAPb_0.85_Ca_0.15_I_3_	1.662	−0.130	1.532
FAPb_0.8_Ca_0.2_I_3_	1.640	−0.128	1.512

## Data Availability

The data presented in this study are available on request from the corresponding authors.

## Supplementary Materials

The following supporting information can be downloaded at: https://www.mdpi.com/article/10.3390/molecules28010372/s1, Figure S1. Three-dimensional (3D) depiction of Be (green), Mg (orange), and Ca (blue)-doped FAPI surfaces related to: (a) (100)-PbI_5_, (b) (100)-PbI_6_, (c) (111)-PbI_3_, and (d) (111)-PbI_6_ terminations. Figure S2. The total density of states for (100) surfaces with doping of (a) 1 atom of Be, Mg, and Ca in PbI_5_ and PbI_6_ terminations, (b) 1-4 atoms of Ca in PbI_6_ and 1-5 atoms of Ca in PbI_5_ terminations, and (c) 1-2 atoms of Mg in PbI_5_ termination. The Fermi level is set at zero eV. Figure S3. The total density of states for (111) surfaces with doping of (a) 1 atom of Be, Mg, and Ca in PbI_3_ and PbI_6_ terminations, (b) 1-4 atoms of Ca in PbI_6_ and 1-5 atoms of Ca in PbI_3_ terminations, and (c) 1-2 atoms of Mg in PbI_3_ termination. The Fermi level is set at zero eV. Figure S4. The real part of dielectric function for (100) and (111) surfaces with doping of (a) 1 atom of Be, Mg, and Ca in all terminations, (b) 1-4 atoms of Ca in both PbI_6_ terminations and 1-5 atoms of Ca in PbI_5_ and PbI_3_ terminations, and (c) 1-2 atoms of Mg in PbI_3_ and PbI_5_ terminations. Figure S5. The imaginary part of dielectric function for (100) and (111) surfaces with doping of (a) 1 atom of Be, Mg, and Ca in all terminations, (b) 1-4 atoms of Ca in both PbI_6_ terminations and 1-5 atoms of Ca in PbI_5_ and PbI_3_ terminations, and (c) 1-2 atoms of Mg in PbI_3_ and PbI_5_ terminations. Figure S6. The refractive index spectra for for (100) and (111) surfaces with doping of (a) 1 atom of Be, Mg, and Ca in all terminations, (b) 1-4 atoms of Ca in both PbI_6_ terminations and 1-5 atoms of Ca in PbI_5_ and PbI_3_ terminations, and (c) 1-2 atoms of Mg in PbI_3_ and PbI_5_ terminations. Figure S7. The extinction coefficient for (100) and (111) surfaces with doping of (a) 1 atom of Be, Mg, and Ca in all terminations, (b) 1-4 atoms of Ca in both PbI_6_ terminations and 1-5 atoms of Ca in PbI_5_ and PbI_3_ terminations, and (c) 1-2 atoms of Mg in PbI_3_ and PbI_5_ terminations. Figure S8. The reflectivity spectra for (100) and (111) surfaces with doping of (a) 1 atom of Be, Mg, and Ca in all terminations, (b) 1-4 atoms of Ca in both PbI_6_ terminations and 1-5 atoms of Ca in PbI_5_ and PbI_3_ terminations, and (c) 1-2 atoms of Mg in PbI_3_ and PbI_5_ terminations. Figure S9. The electron energy loss function versus photon energy for (100) and (111) surfaces with doping of (a) 1 atom of Be, Mg, and Ca in all terminations, (b) 1-4 atoms of Ca in both PbI_6_ terminations and 1-5 atoms of Ca in PbI_5_ and PbI_3_ terminations, and (c) 1-2 atoms of Mg in PbI_3_ and PbI_5_ terminations.

## Sample Availability

Samples of the compounds are not available from the authors.
