# Supplementary material for: A DFT Study of Alkaline Earth Metal-Doped FAPbI3 (111) and (100) Surfaces
Source: Molecules. 2023 Jan 2;28(1):372. doi: 10.3390/molecules28010372 (PMC9822229; doi:10.3390/molecules28010372)
Supplement: Supplementary file 1 [file molecules-28-00372-s001.zip › molecules-2089209-supplementary.pdf]

Supporting Information File

A DFT Study of Alkaline Earth Metal-Doped $FAPbI_3$ (111) and (100) Surfaces

Maryam RaeisianAsl [1], Saeedeh Sarabadani Tafreshi [1,*] and Nora H. de Leeuw [2,3,*]

[1] Department of Chemistry, Amirkabir University of Technology, No. 350, Hafez Avenue, Valiasr Square, Tehran 1591634311, Iran
[2] School of Chemistry, University of Leeds, Leeds LT2 9JT, UK
[3] Department of Earth Sciences, Utrecht University, 3584 CB Utrecht, The Netherlands
* Correspondence: s.s.tafreshi@aut.ac.ir (S.S.T.); n.h.deleeuw@leeds.ac.uk (N.H.d.L.)

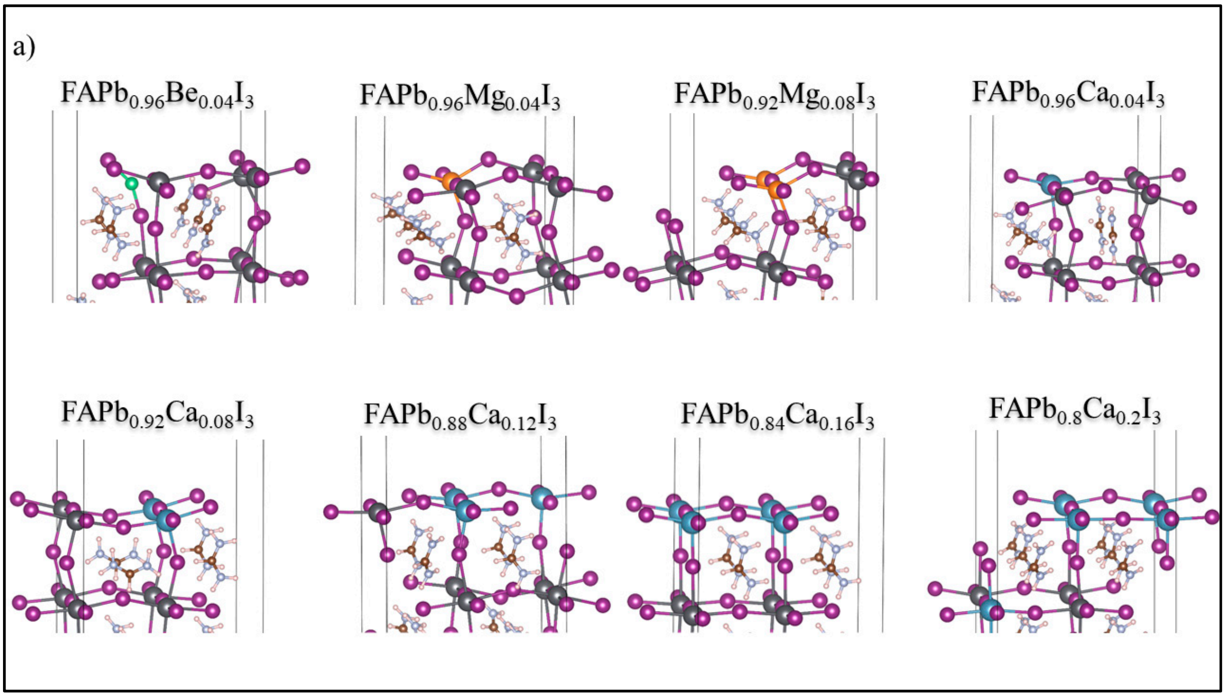

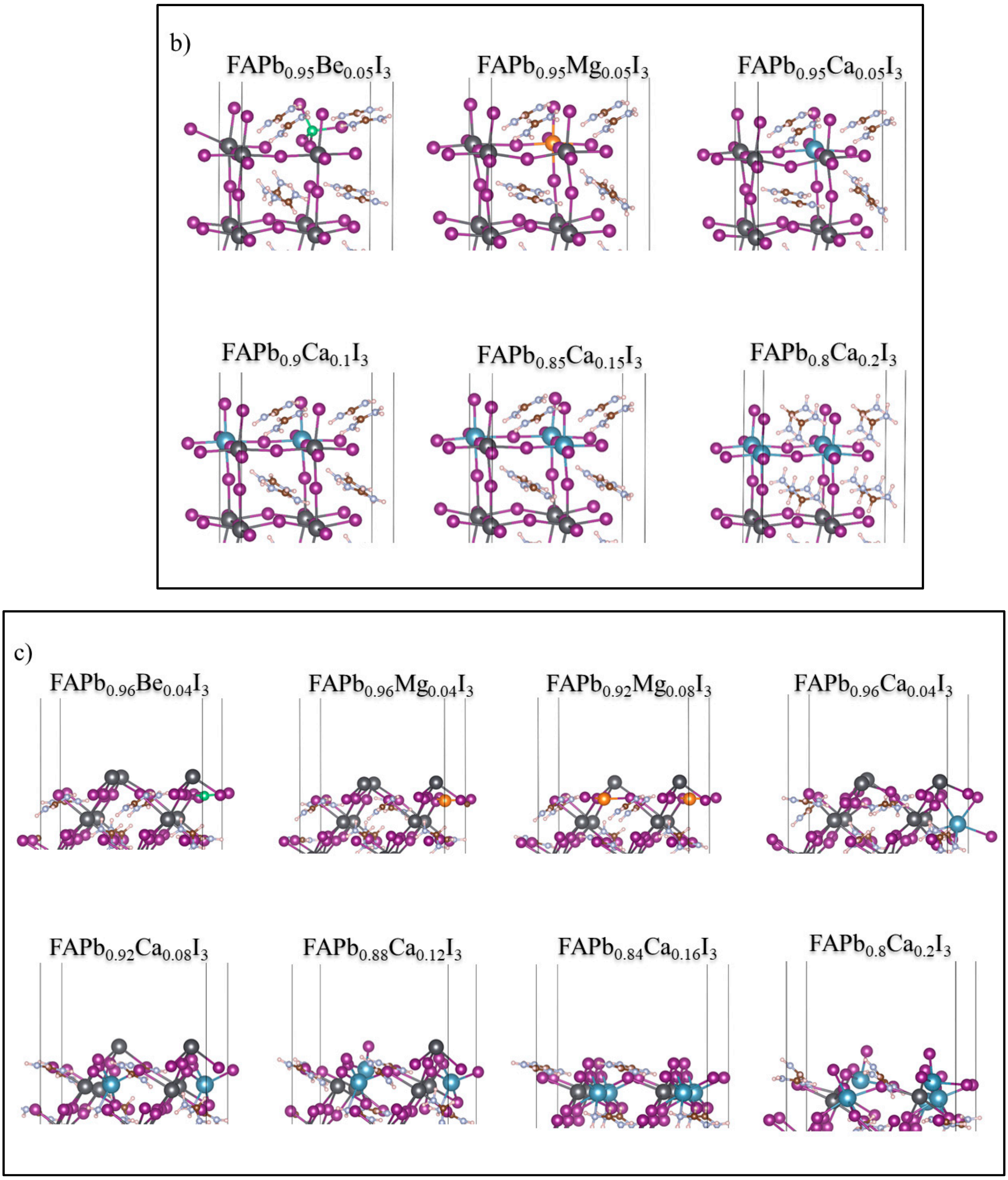
b)
$FAPb_{0.95}Be_{0.05}I_3$
$FAPb_{0.95}Mg_{0.05}I_3$
$FAPb_{0.95}Ca_{0.05}I_3$
$FAPb_{0.9}Ca_{0.1}I_3$
$FAPb_{0.85}Ca_{0.15}I_3$
$FAPb_{0.8}Ca_{0.2}I_3$
c)
$FAPb_{0.96}Be_{0.04}I_3$
$FAPb_{0.96}Mg_{0.04}I_3$
$FAPb_{0.92}Mg_{0.08}I_3$
$FAPb_{0.96}Ca_{0.04}I_3$
$FAPb_{0.92}Ca_{0.08}I_3$
$FAPb_{0.88}Ca_{0.12}I_3$
$FAPb_{0.84}Ca_{0.16}I_3$
$FAPb_{0.8}Ca_{0.2}I_3$

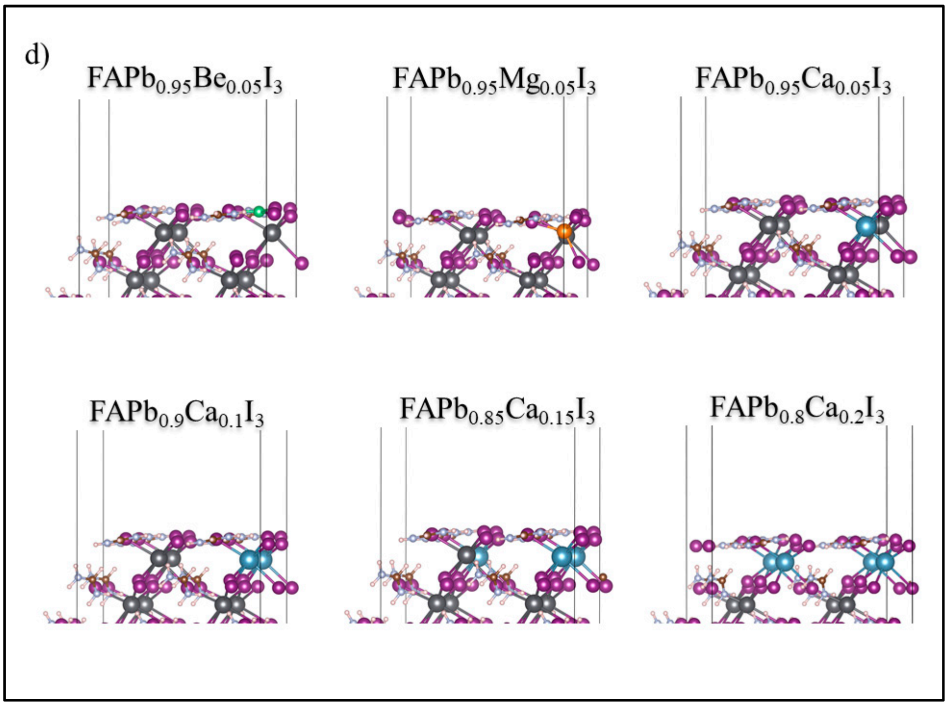

Figure S1. Three-dimensional (3D) depiction of Be (green), Mg (orange), and Ca (blue)-doped FAPI surfaces related to: a) (100)-PbI_5, b) (100)-PbI_6, c) (111)-PbI_3, and d) (111)-PbI_6 terminations.

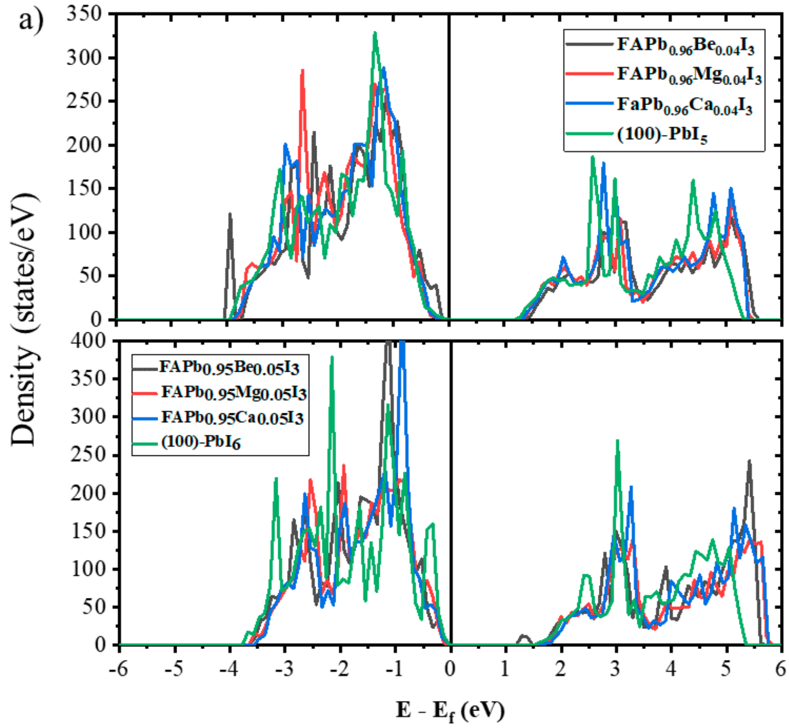

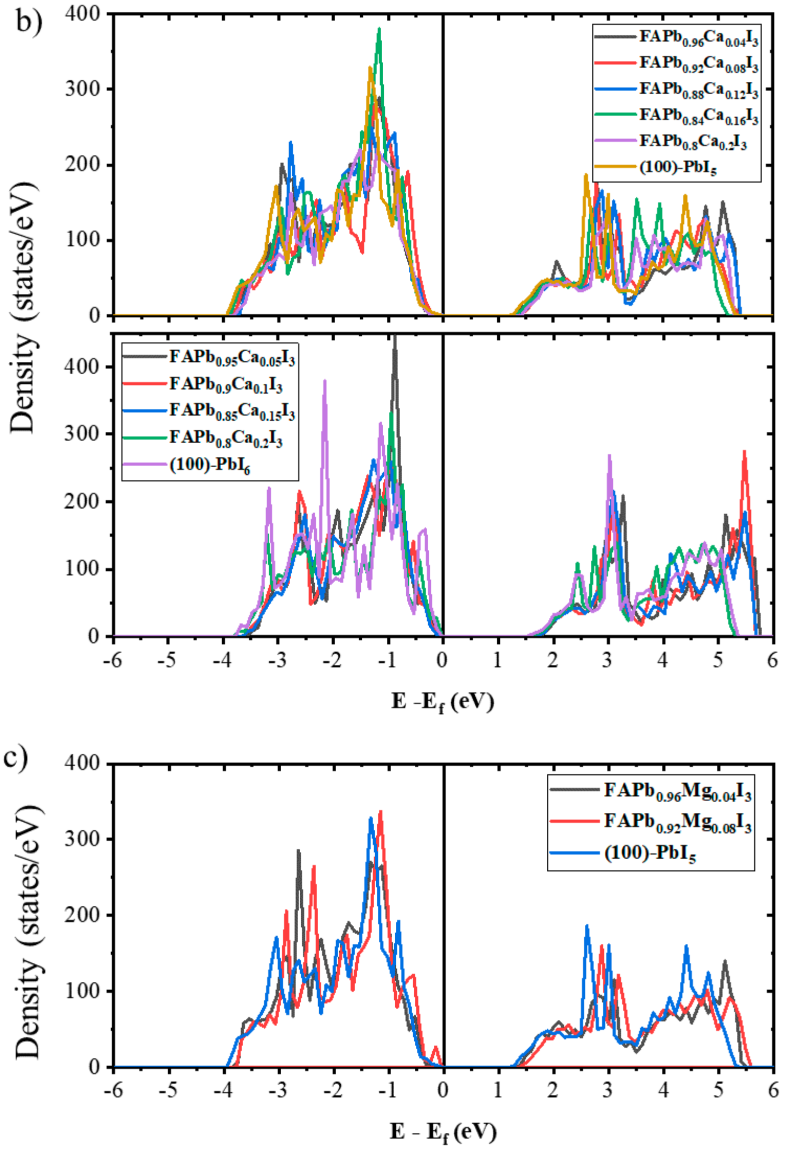

Figure S2. The total density of states for (100) surfaces with doping of a) 1 atom of Be, Mg, and Ca in PbI_5 and PbI_6 terminations, b) 1-4 atoms of Ca in PbI_6 and 1-5 atoms of Ca in PbI_5 terminations, and c) 1-2 atoms of Mg in PbI_5 termination. The Fermi level is set at zero eV.

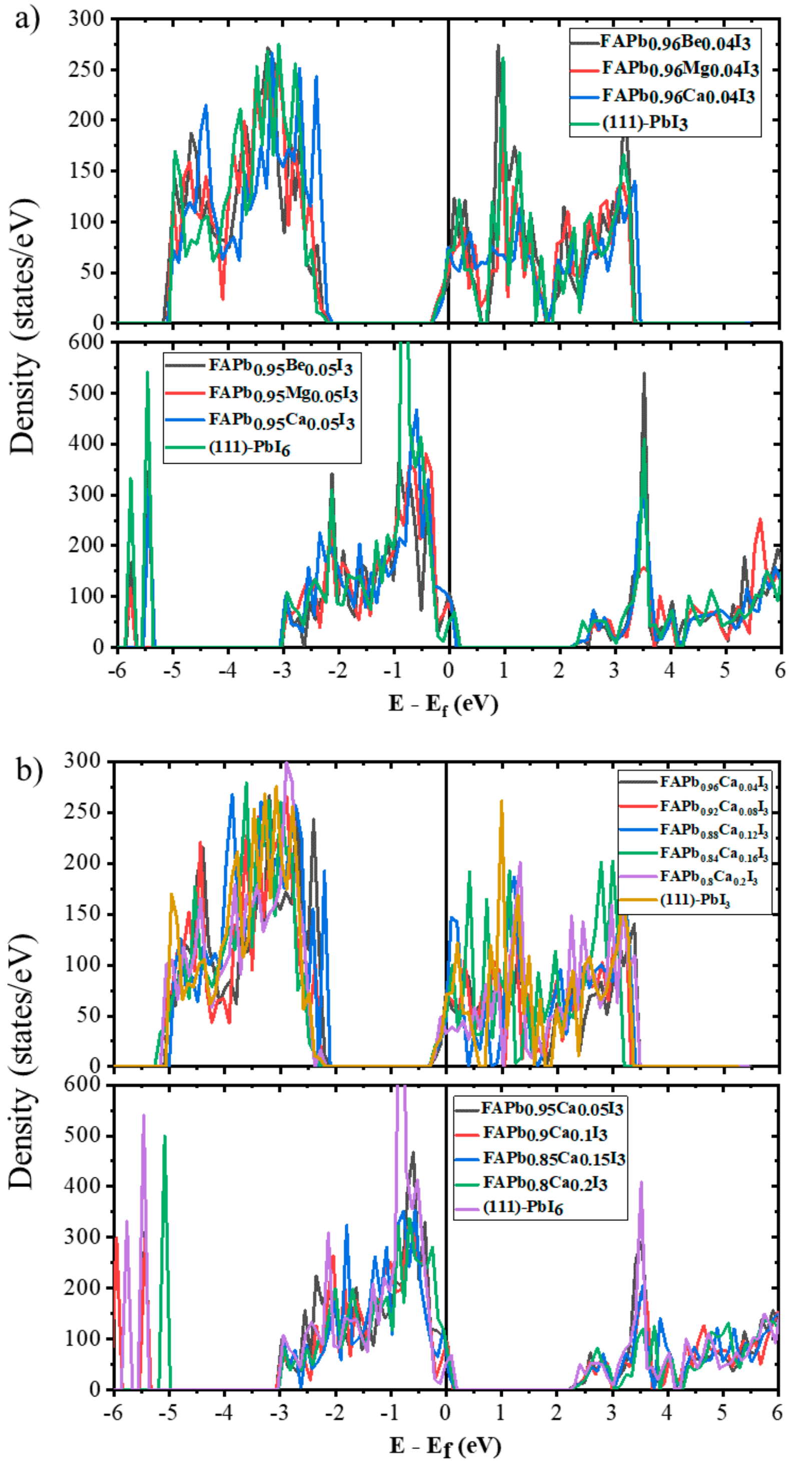
a)
FAPb0.96Be0.04I3
FAPb0.96Mg0.04I3
FAPb0.96Ca0.04I3
(111)-PbI3
FAPb0.95Be0.05I3
FAPb0.95Mg0.05I3
FAPb0.95Ca0.05I3
(111)-PbI6
Density (states/eV)
E - Ef (eV)
b)
FAPb0.96Ca0.04I3
FAPb0.92Ca0.08I3
FAPb0.88Ca0.12I3
FAPb0.84Ca0.16I3
FAPb0.8Ca0.2I3
(111)-PbI3
FAPb0.95Ca0.05I3
FAPb0.9Ca0.1I3
FAPb0.85Ca0.15I3
FAPb0.8Ca0.2I3
(111)-PbI6
Density (states/eV)
E - Ef (eV)

c)

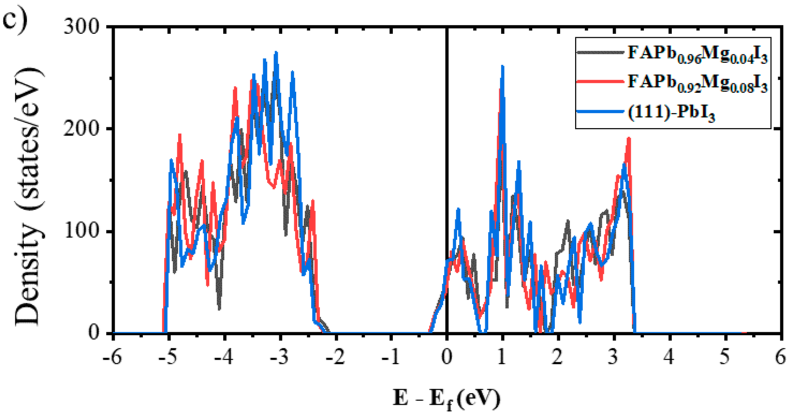

Figure S3. The total density of states for (111) surfaces with doping of a) 1 atom of Be, Mg, and Ca in PbI_3 and PbI_6 terminations, b) 1-4 atoms of Ca in PbI_6 and 1-5 atoms of Ca in PbI_3 terminations, and c) 1-2 atoms of Mg in PbI_3 termination. The Fermi level is set at zero eV.

a)

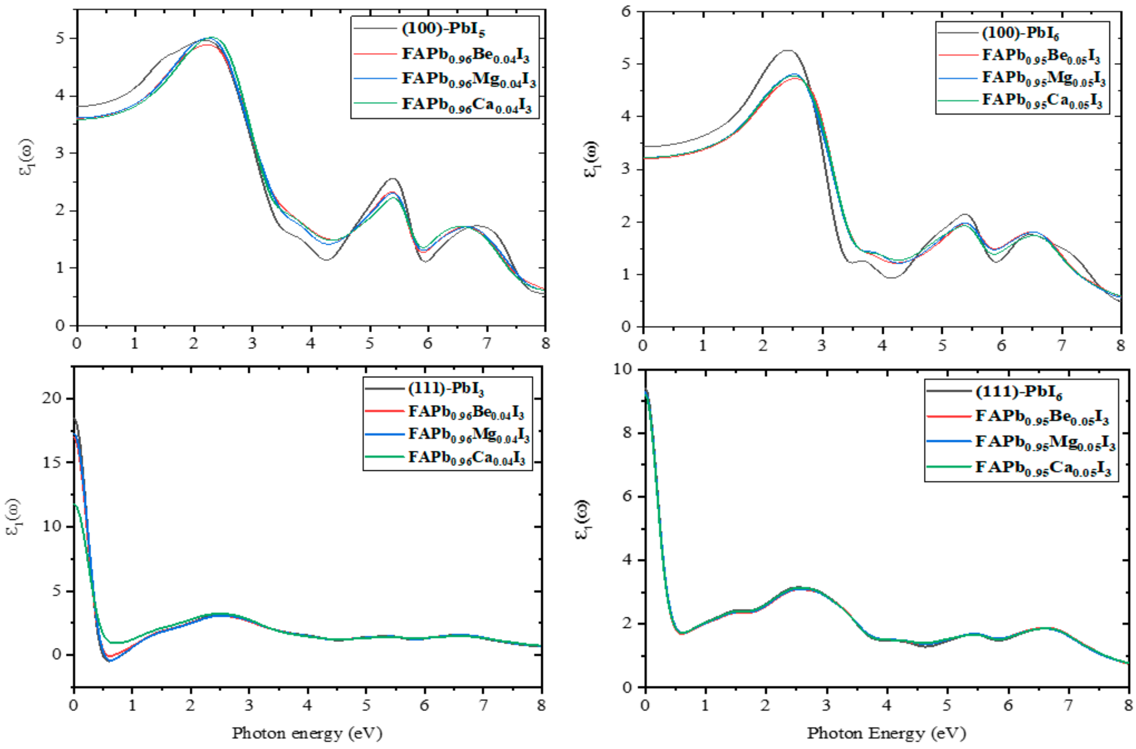

b)

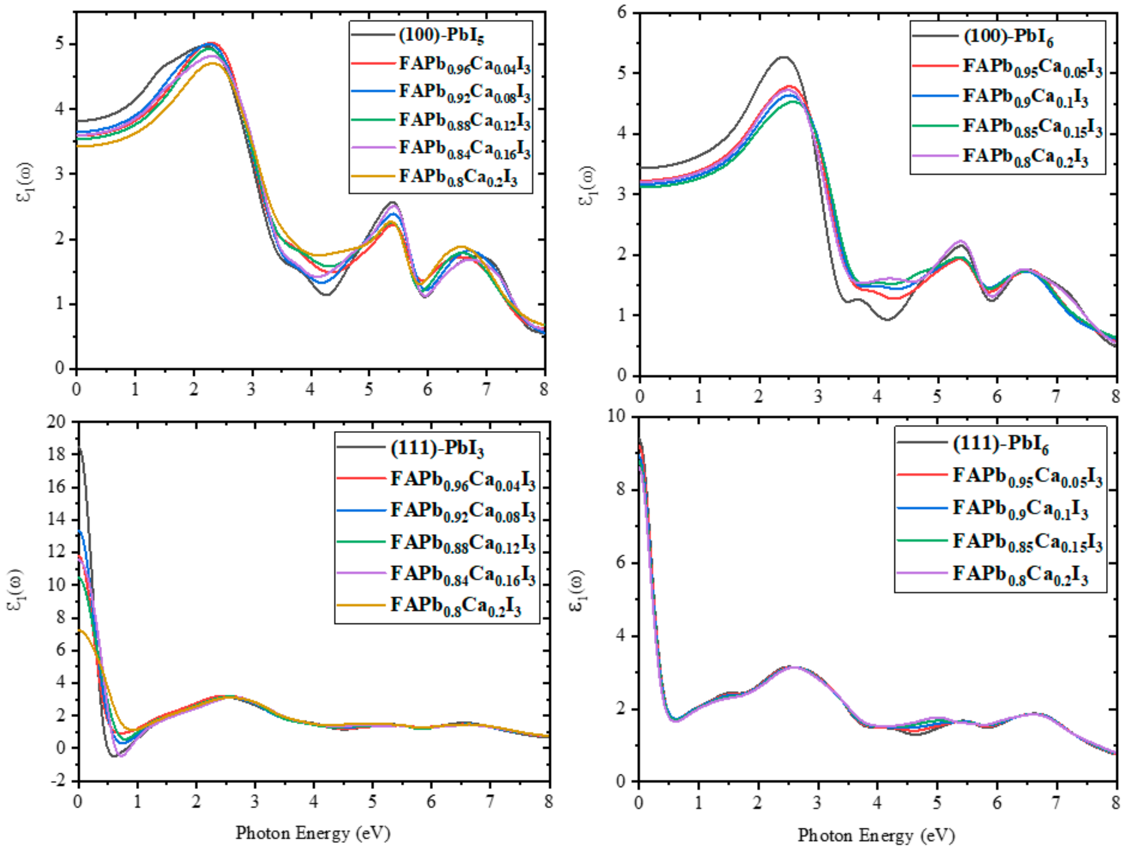

c)

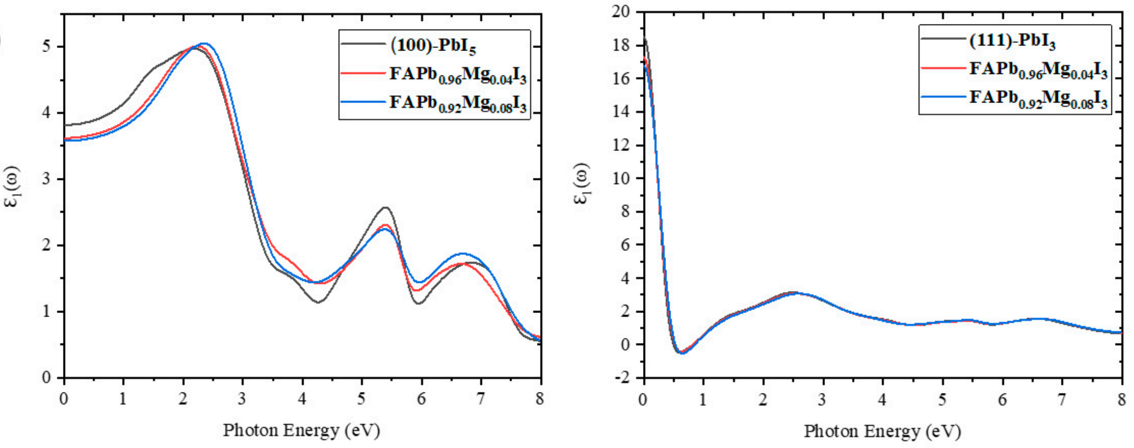

Figure S4. The real part of dielectric function for (100) and (111) surfaces with doping of a) 1 atom of Be, Mg, and Ca in all terminations, b) 1-4 atoms of Ca in both PbI_6 terminations and 1-5 atoms of Ca in PbI_5 and PbI_3 terminations, and c) 1-2 atoms of Mg in PbI_3 and PbI_5 terminations.

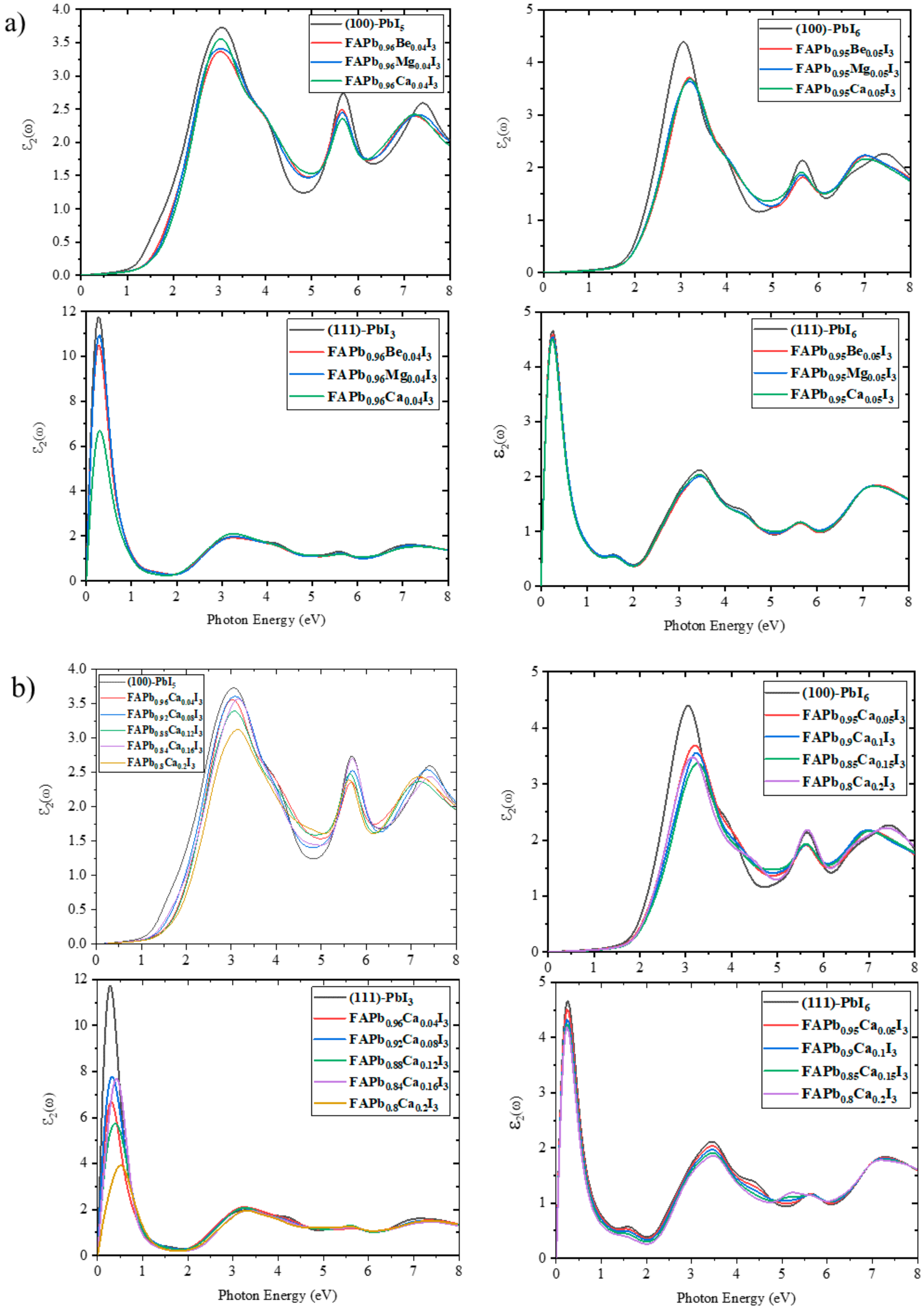

a)
(100)-PbI5
FAPb0.96Be0.04I3
FAPb0.96Mg0.04I3
FAPb0.96Ca0.04I3
(100)-PbI6
FAPb0.95Be0.05I3
FAPb0.95Mg0.05I3
FAPb0.95Ca0.05I3
(111)-PbI3
(111)-PbI6
ε2(ω)
Photon Energy (eV)
b)
FAPb0.92Ca0.08I3
FAPb0.88Ca0.12I3
FAPb0.84Ca0.16I3
FAPb0.8Ca0.2I3
FAPb0.9Ca0.1I3
FAPb0.85Ca0.15I3

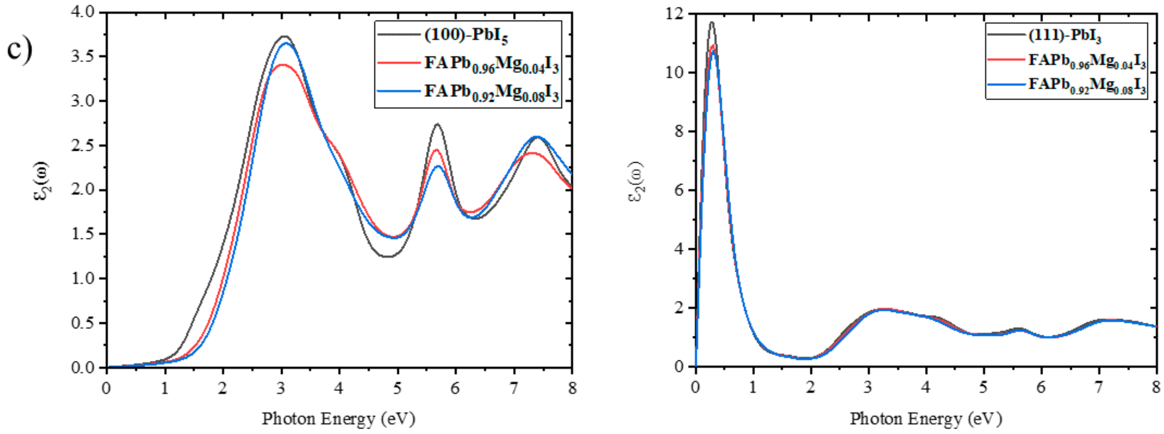

Figure S5. The imaginary part of dielectric function for (100) and (111) surfaces with doping of a) 1 atom of Be, Mg, and Ca in all terminations, b) 1-4 atoms of Ca in both PbI_6 terminations and 1-5 atoms of Ca in PbI_5 and PbI_3 terminations, and c) 1-2 atoms of Mg in PbI_3 and PbI_5 terminations.

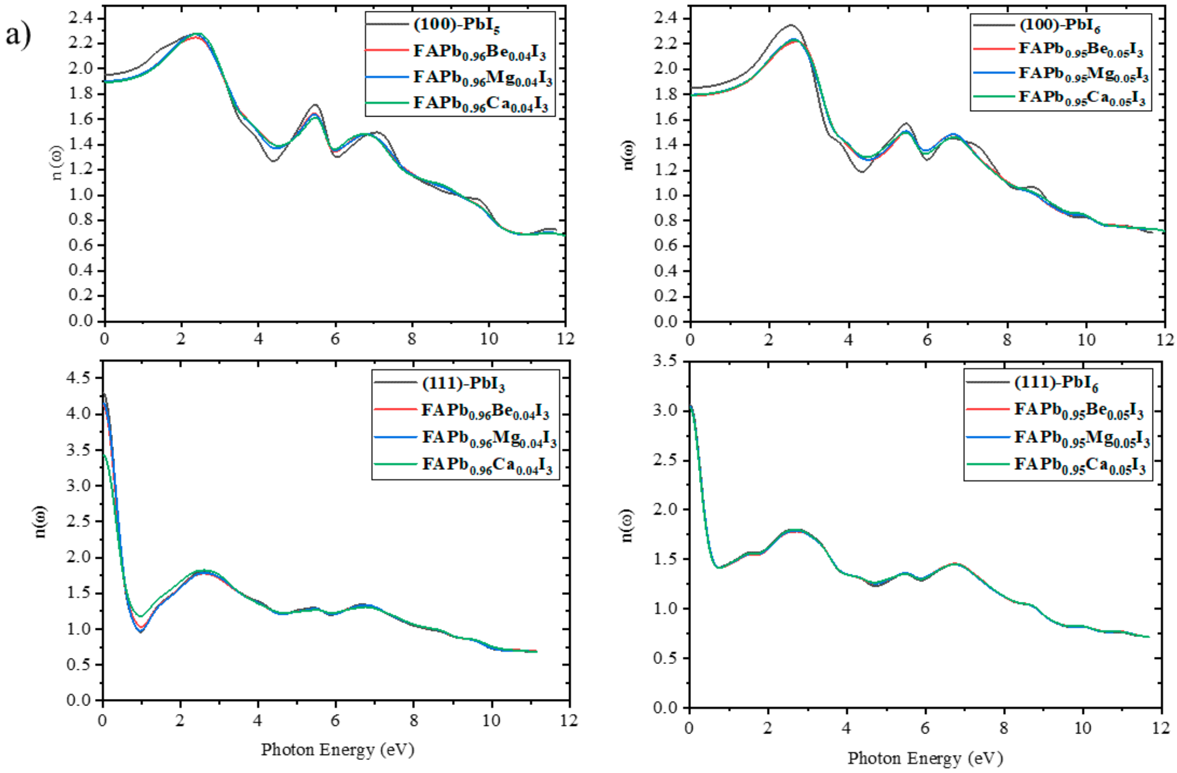

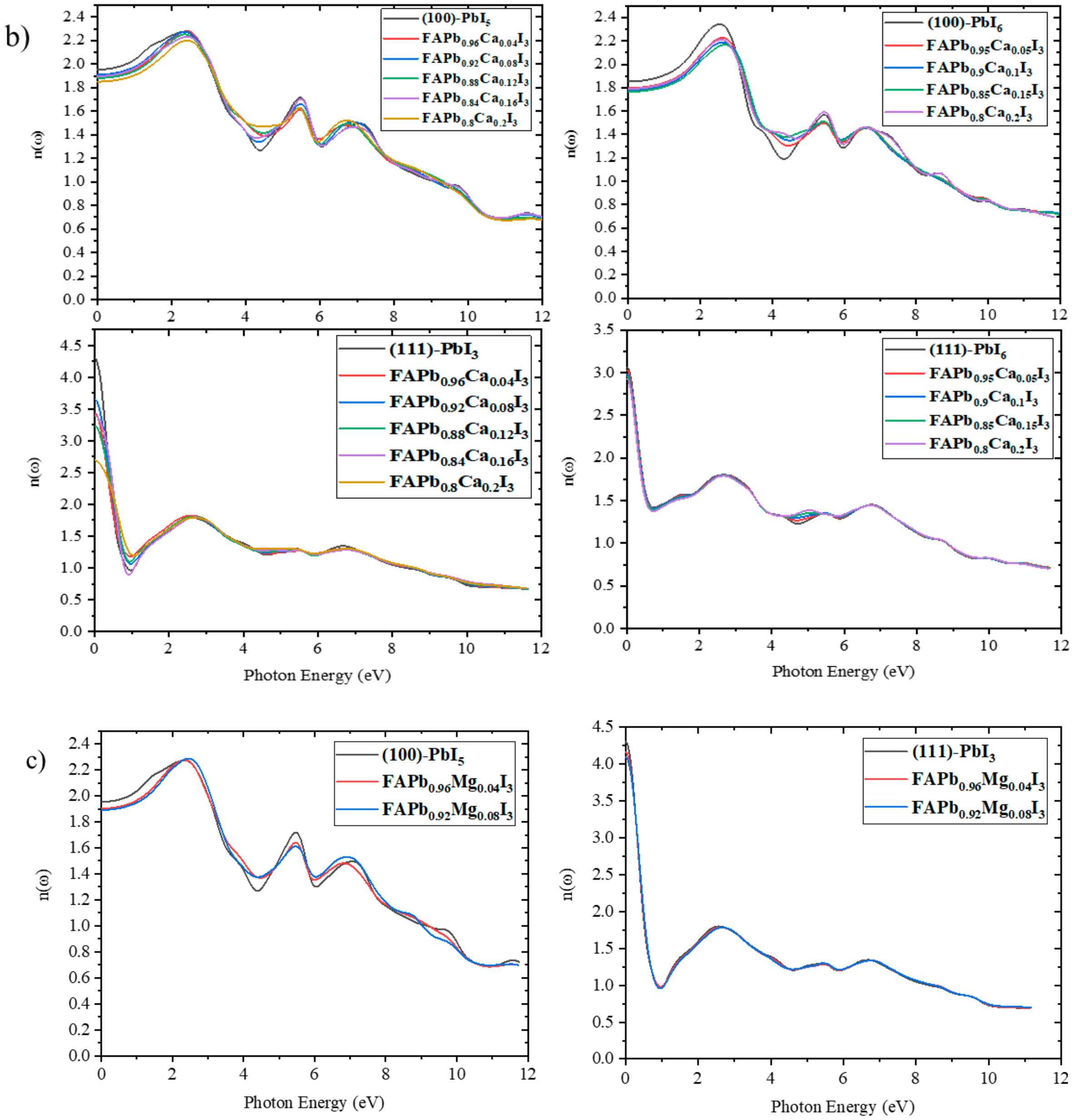

Figure S6. The refractive index spectra for for (100) and (111) surfaces with doping of a) 1 atom of Be, Mg, and Ca in all terminations, b) 1-4 atoms of Ca in both PbI_6 terminations and 1-5 atoms of Ca in PbI_5 and PbI_3 terminations, and c) 1-2 atoms of Mg in PbI_3 and PbI_5 terminations.

a)

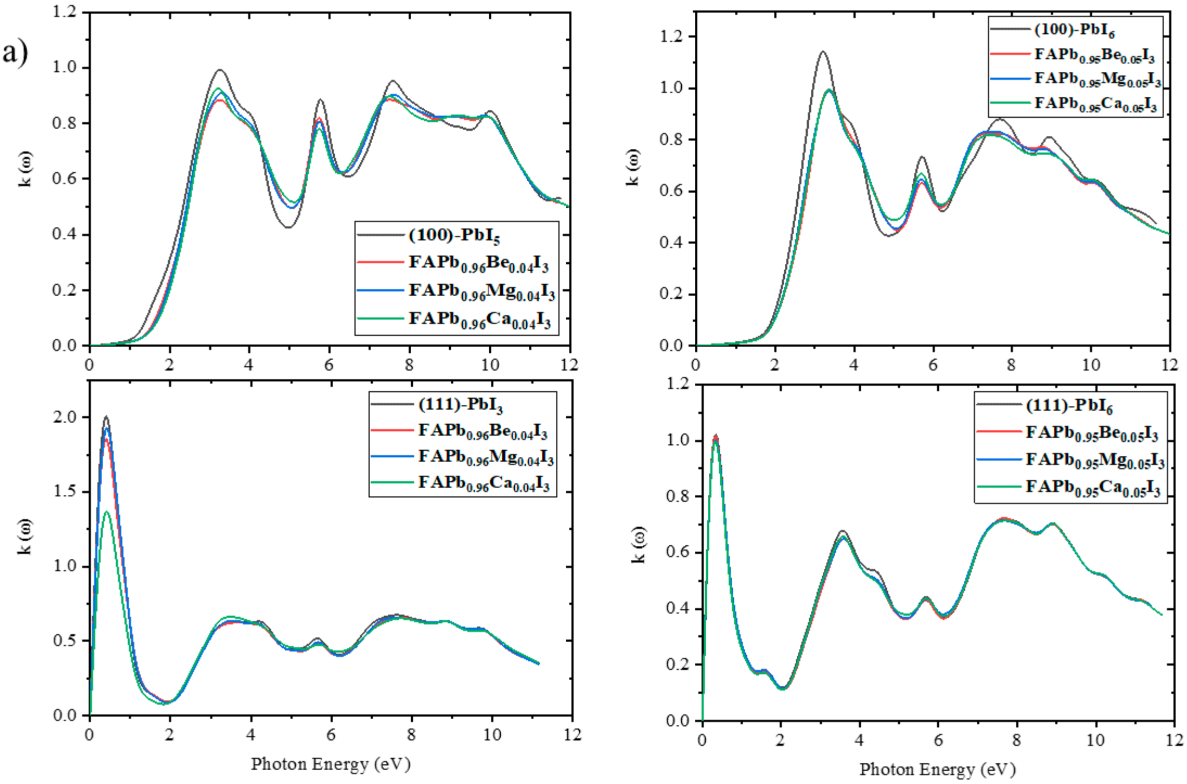

k (ω)
Photon Energy (eV)
(100)-PbI_5
$FAPb_{0.96}Be_{0.04}I_3$
$FAPb_{0.96}Mg_{0.04}I_3$
$FAPb_{0.96}Ca_{0.04}I_3$
(100)-PbI_6
$FAPb_{0.95}Be_{0.05}I_3$
$FAPb_{0.95}Mg_{0.05}I_3$
$FAPb_{0.95}Ca_{0.05}I_3$
(111)-PbI_3
$FAPb_{0.96}Be_{0.04}I_3$
$FAPb_{0.96}Mg_{0.04}I_3$
$FAPb_{0.96}Ca_{0.04}I_3$
(111)-PbI_6
$FAPb_{0.95}Be_{0.05}I_3$
$FAPb_{0.95}Mg_{0.05}I_3$
$FAPb_{0.95}Ca_{0.05}I_3$

b)

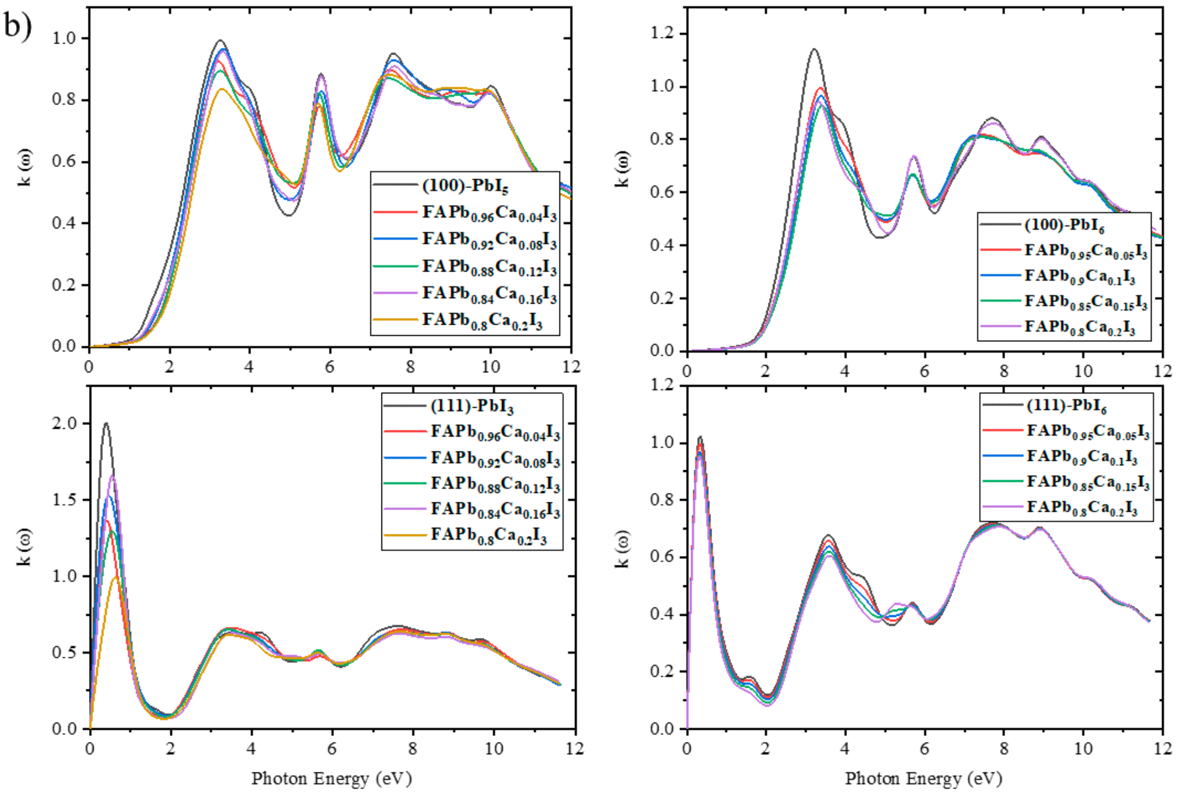

k (ω)
Photon Energy (eV)
(100)-PbI_5
$FAPb_{0.96}Ca_{0.04}I_3$
$FAPb_{0.92}Ca_{0.08}I_3$
$FAPb_{0.88}Ca_{0.12}I_3$
$FAPb_{0.84}Ca_{0.16}I_3$
$FAPb_{0.8}Ca_{0.2}I_3$
(100)-PbI_6
$FAPb_{0.95}Ca_{0.05}I_3$
$FAPb_{0.9}Ca_{0.1}I_3$
$FAPb_{0.85}Ca_{0.15}I_3$
$FAPb_{0.8}Ca_{0.2}I_3$
(111)-PbI_3
$FAPb_{0.96}Ca_{0.04}I_3$
$FAPb_{0.92}Ca_{0.08}I_3$
$FAPb_{0.88}Ca_{0.12}I_3$
$FAPb_{0.84}Ca_{0.16}I_3$
$FAPb_{0.8}Ca_{0.2}I_3$
(111)-PbI_6
$FAPb_{0.95}Ca_{0.05}I_3$
$FAPb_{0.9}Ca_{0.1}I_3$
$FAPb_{0.85}Ca_{0.15}I_3$
$FAPb_{0.8}Ca_{0.2}I_3$

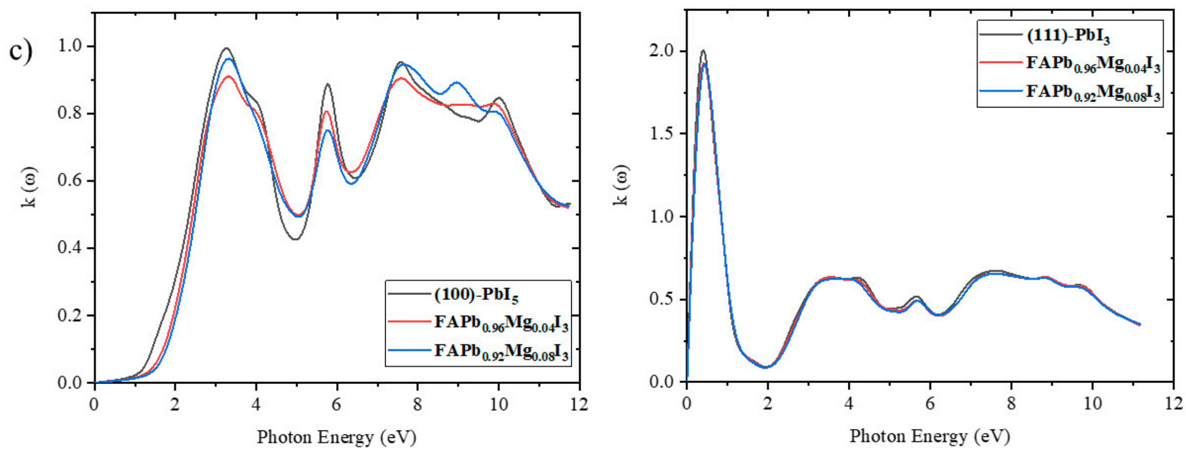

Figure S7. The extinction coefficient for (100) and (111) surfaces with doping of a) 1 atom of Be, Mg, and Ca in all terminations, b) 1-4 atoms of Ca in both PbI_6 terminations and 1-5 atoms of Ca in PbI_5 and PbI_3 terminations, and c) 1-2 atoms of Mg in PbI_3 and PbI_5 terminations.

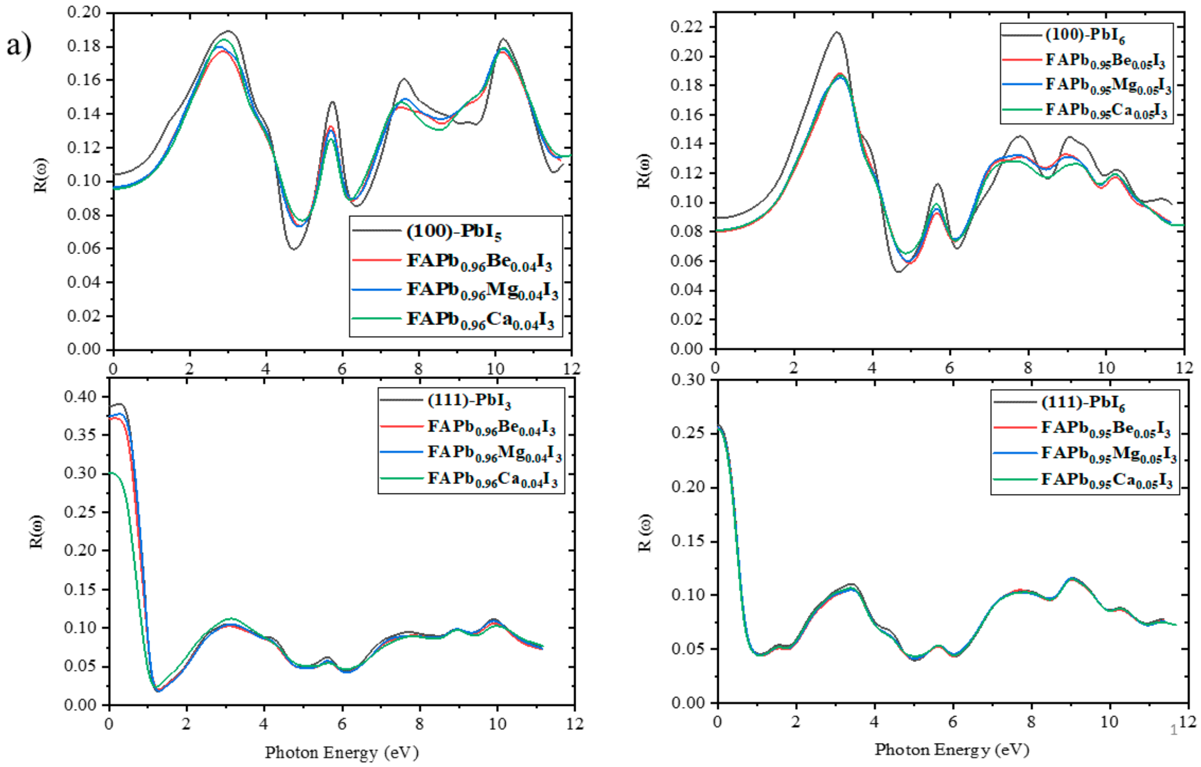

b)

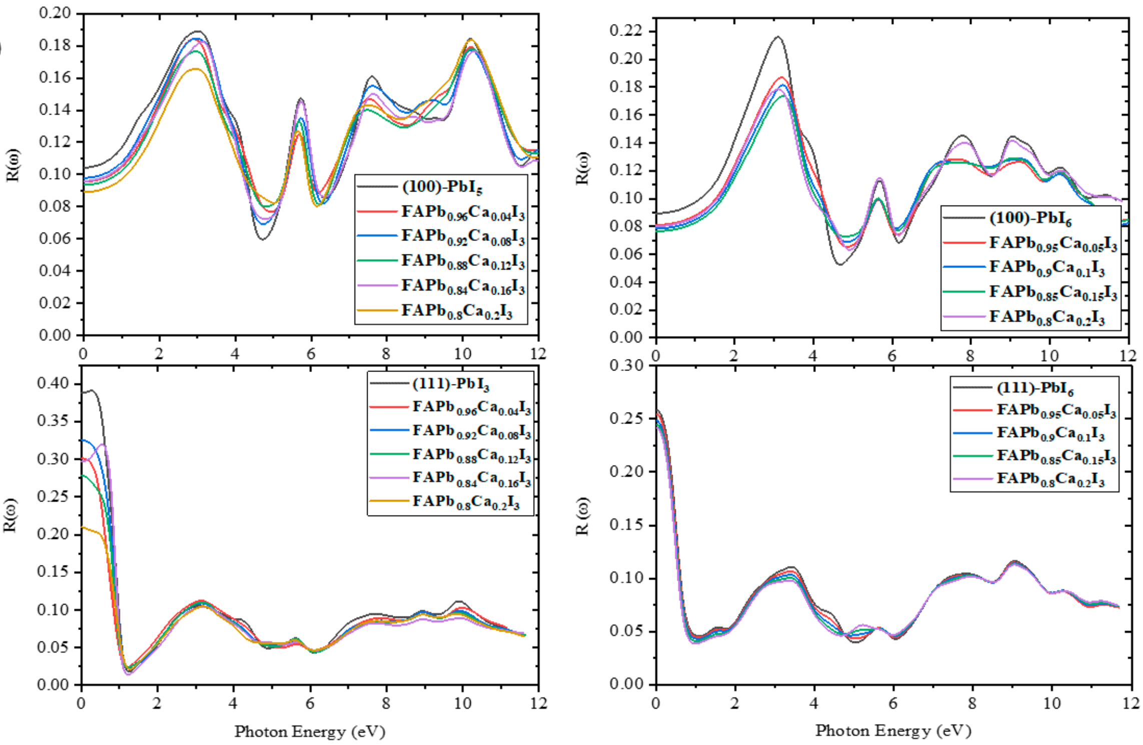

c)

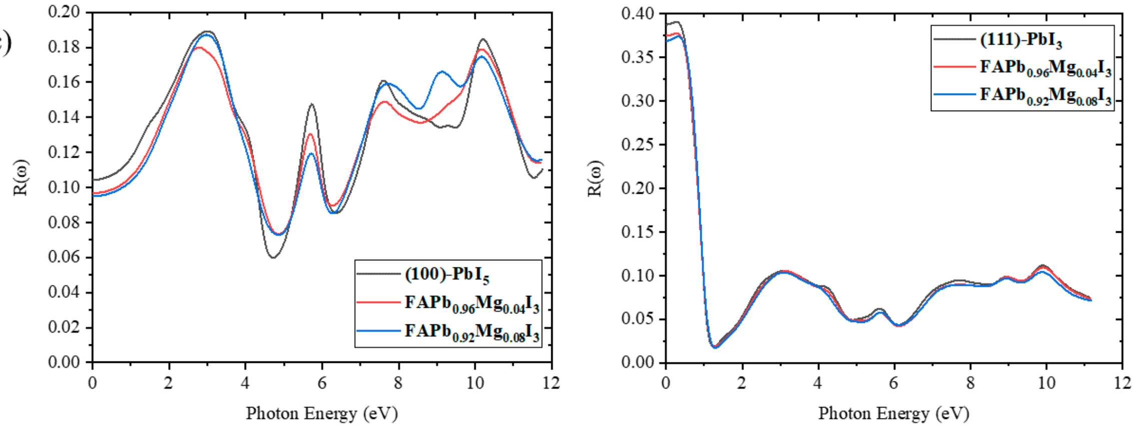

Figure S8. The reflectivity spectra for (100) and (111) surfaces with doping of a) 1 atom of Be, Mg, and Ca in all terminations, b) 1-4 atoms of Ca in both PbI_6 terminations and 1-5 atoms of Ca in PbI_5 and PbI_3 terminations, and c) 1-2 atoms of Mg in PbI_3 and PbI_5 terminations.

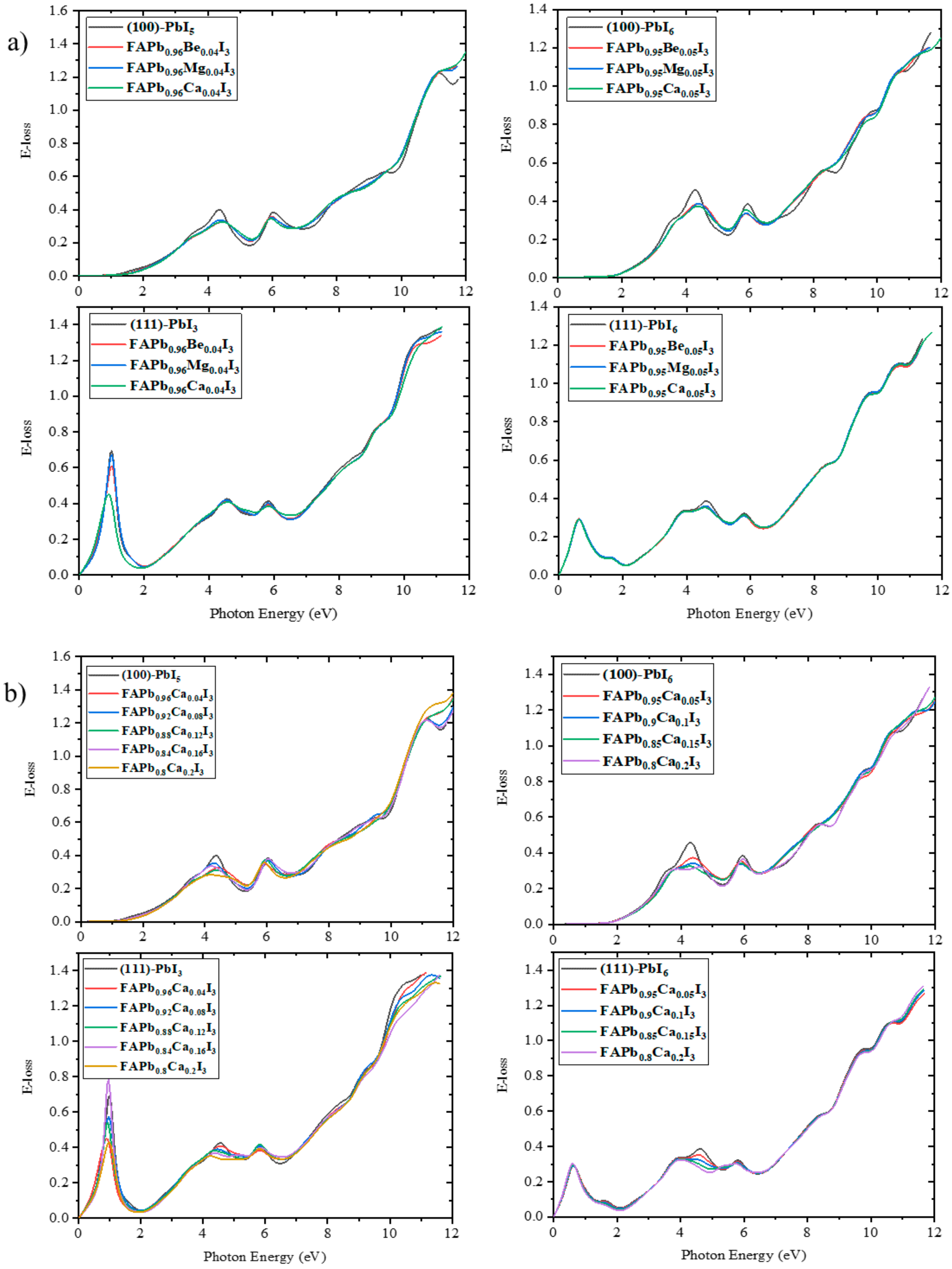
a)
(100)-PbI5
FAPb0.96Be0.04I3
FAPb0.96Mg0.04I3
FAPb0.96Ca0.04I3
(100)-PbI6
FAPb0.95Be0.05I3
FAPb0.95Mg0.05I3
FAPb0.95Ca0.05I3
(111)-PbI3
FAPb0.96Be0.04I3
FAPb0.96Mg0.04I3
FAPb0.96Ca0.04I3
(111)-PbI6
FAPb0.95Be0.05I3
FAPb0.95Mg0.05I3
FAPb0.95Ca0.05I3
E-loss
Photon Energy (eV)
b)
(100)-PbI5
FAPb0.96Ca0.04I3
FAPb0.92Ca0.08I3
FAPb0.88Ca0.12I3
FAPb0.84Ca0.16I3
FAPb0.8Ca0.2I3
(100)-PbI6
FAPb0.95Ca0.05I3
FAPb0.9Ca0.1I3
FAPb0.85Ca0.15I3
FAPb0.8Ca0.2I3
(111)-PbI3
FAPb0.96Ca0.04I3
FAPb0.92Ca0.08I3
FAPb0.88Ca0.12I3
FAPb0.84Ca0.16I3
FAPb0.8Ca0.2I3
(111)-PbI6
FAPb0.95Ca0.05I3
FAPb0.9Ca0.1I3
FAPb0.85Ca0.15I3
FAPb0.8Ca0.2I3
E-loss
Photon Energy (eV)

c)

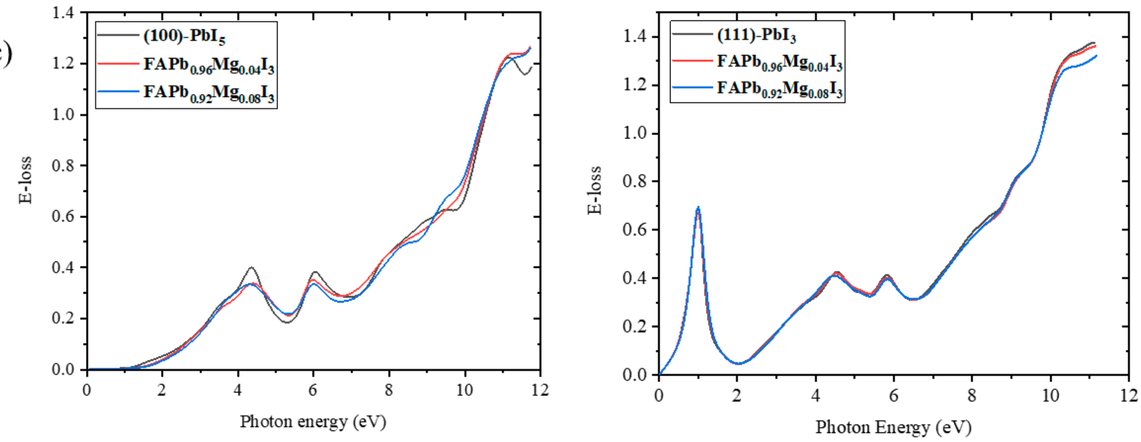

Figure S9. The electron energy loss function versus photon energy for (100) and (111) surfaces with doping of a) 1 atom of Be, Mg, and Ca in all terminations, b) 1-4 atoms of Ca in both PbI_6 terminations and 1-5 atoms of Ca in PbI_5 and PbI_3 terminations, and c) 1-2 atoms of Mg in PbI_3 and PbI_5 terminations.
